# Co-delivery of targeted hypoallergens and resiquimod powders using silk fibroin microneedles for effective allergen-specific immunotherapy

**DOI:** 10.7150/thno.114152

**Published:** 2025-07-24

**Authors:** Zhen Wang, Xiaolei Lu, Lingzhi Wu, Xin Jiang, Wei Wang, Yahua Wang

**Affiliations:** 1 Department of Pharmacy, Affiliated Hospital of Jiaxing University, The First Hospital of Jiaxing, Jiaxing, 314000, China.; 2 College of Materials and Textile Engineering, Jiaxing University, Jiaxing 314001, China.

**Keywords:** allergen-specific immunotherapy (AIT), powder-laden microneedles, mannan-ovalbumin (mOVA) conjugates, resiquimod (R848), allergic asthma

## Abstract

**Rationale:** The cutaneous route exploits the immunocompetence of the skin, making it a favourable route for allergen-specific immunotherapy (AIT), but there must be a balance between minimal skin disruption and precise allergen delivery. Thus, we propose the use of powder-laden microneedles (pMNs) for the sustained epidermal delivery of powdery hypoallergens and immunomodulators.

**Methods:** In this study, targeted hypoallergenic derivatives, namely, mannan-ovalbumin (mOVA) conjugates, were synthesized in a single-step process. For minimally invasive self-administration into the skin, pMNs were constructed using highly biocompatible silk fibroin (SF) with a cavity in the basal portion of each needle, which was filled with lyophilized mOVA and resiquimod powders (mOVA/R848 pMN). The resulting mOVA/R848 pMN arrays were thoroughly examined for their physical morphology, mechanical property, accumulation ability, and immune profile.

**Results:** The deposition of powdery mOVA and R848 improved uptake by skin dendritic cells (DCs) and stimulated the immune system more than 10 d after application. On the basis of these features, mOVA/R848 pMN arrays increased the induction of protective immune mechanisms to suppress Th2 immunity, including the Treg/Th1 bias, as well as the production of anti-inflammatory cytokines and neutralizing antibodies in an asthmatic mouse model. Compared with traditional subcutaneous immunotherapy (SCIT), a total dose of only 75 μg of OVA over three treatments was sufficient to restore immune balance and alleviate allergic symptoms during allergen exposure, highlighting the dose-sparing and frequency-sparing potential of mOVA/R848 pMN.

**Conclusion:** Optimal mOVA/R848 pMN arrays represent a new therapeutic strategy for allergy treatment with convenient administration and satisfactory outcomes.

## Introduction

Immunoglobulin E (IgE)-mediated allergic diseases affect approximately 20% of the global population, thereby imposing a global medical and economic burden 1. Allergen-specific immunotherapy (AIT) is the only curative treatment for allergic diseases, and it involves the establishment of allergen tolerance through repetitive administration of antigens in their naked form 2. Despite the proven clinical efficacy of standard AIT administered via subcutaneous injection (SCIT) or sublingual application (SLIT), fewer than 5% of patients decide to undergo this time-consuming and laborious procedure, with concerns about its efficacy and safety 3. To address these challenges, the use of allergen derivatives combined with novel adjuvants and alternative routes of administration have been shown to increase efficacy while decreasing unwanted adverse effects 4.

Rational allergen modification for targeting skin-resident dendritic cells (DCs) is an attractive approach because these DCs are indispensable for the development of allergy or tolerance 5. Mannan is a yeast polysaccharide that activates C-type lectin receptors (CLRs), which are pattern recognition receptors normally expressed on DCs. Coupling mannan to allergens has improved uptake and efficient presentation by DCs in a process dependent on both mannose receptor (MR)-mediated and DC-specific intercellular adhesion molecule 3-grabbing nonintegrin (DC-SIGN)-mediated internalization. These targeted derivatives exhibit enhanced trafficking to draining lymph nodes, where B and T lymphocyte activation are induced to trigger effective cellular and humoral immunity 6. Thus, mannan decoration can be used to optimize AIT because of the potential of mannan as an adjuvant to enhance allergen capture and overall immune responses, as observed in phase II/III studies, which are currently ongoing 7.

Adjuvants are substances capable of increasing the immunogenicity of coapplied allergens and inducing the desired immune activation. Unlike conventional aluminium salts, adjuvants recognized by Toll-like receptors (TLRs) are specific immunomodulators that favour anti-allergic T lymphocyte responses 8. Resiquimod (R848), a TLR7/8 agonist for topical use, counteracts Th2-dominant hypersensitivity by upregulating Th1 polarization, inducing regulatory T cells (Tregs) in tissues, and inhibiting IgE production by acting directly on B lymphocytes 9, 10. Because AIT is known to be beneficial, combining allergens/adjuvants within a delivery platform is desirable for shortening treatment schedules while simultaneously providing a high safety profile and patient acceptance 11.

Cutaneous administration exploits the immunocompetence of the skin, representing a promising route for AIT 12. Epicutaneous immunotherapy (EPIT) leverages the capacity of skin-resident DCs, particularly Langerhans cells (LCs) in the superficial skin layers, to take up allergens for the induction of immune tolerance, with varying degrees of success in phase II/III clinical trials 13, 14. The Viaskin delivery system is designed to facilitate the diffusion of powdery allergens into the active epidermis, but the stratum corneum (SC) barrier impedes allergen delivery via intact skin, thus explaining less consistent outcomes across various skin types independent of age and ethnicity 15. Because excessively damaged skin predisposes individuals to the development of allergic inflammation, the sufficient and precise quantification of the allergens delivered into the epidermis remains a major challenge.

Microneedles (MNs) are micron-sized arrays of needles that can achieve an ideal balance of minimal skin disruption while ensuring high consistency, efficiency and reliability in the co-delivery of antigens and immunostimulants into the epidermal and dermal layers of skin 16, 17. Moreover, advances in biomaterials may endow MNs with smart functions such as stimuli responsiveness and sustained release 18, 19. Compared with ablative fractional lasers, MNs provide efficient immune modulation in a painless and minimally invasive fashion that can be broadly practised in the clinic and at home 20, 21. In previous attempts at MN-aided allergen delivery, stainless-steel, dip-coated MNs and dissolving MNs have been widely utilized to treat respiratory and food allergies in mouse models 22, 23. Nevertheless, the substantial loss of antigenicity or allergenicity during the repeated process of dipping and drying is one of the major drawbacks associated with coated and dissolving MNs. Although the initial idea of MN-based AIT was derived from early results in epicutaneous administration, preclinical testing with mouse models has shown that not all therapeutic agents localize in the epidermis and that a significant fraction is delivered into the dermis because these MNs range in size to more than 700 μm in length 12, 24. Therefore, MN arrays should be rationally designed for application purposes, considering their structural parameters, such as shape and size, thus enabling the targeted delivery of bioactive substances to the epidermis and superficial dermis 25.

**Figure 1:**
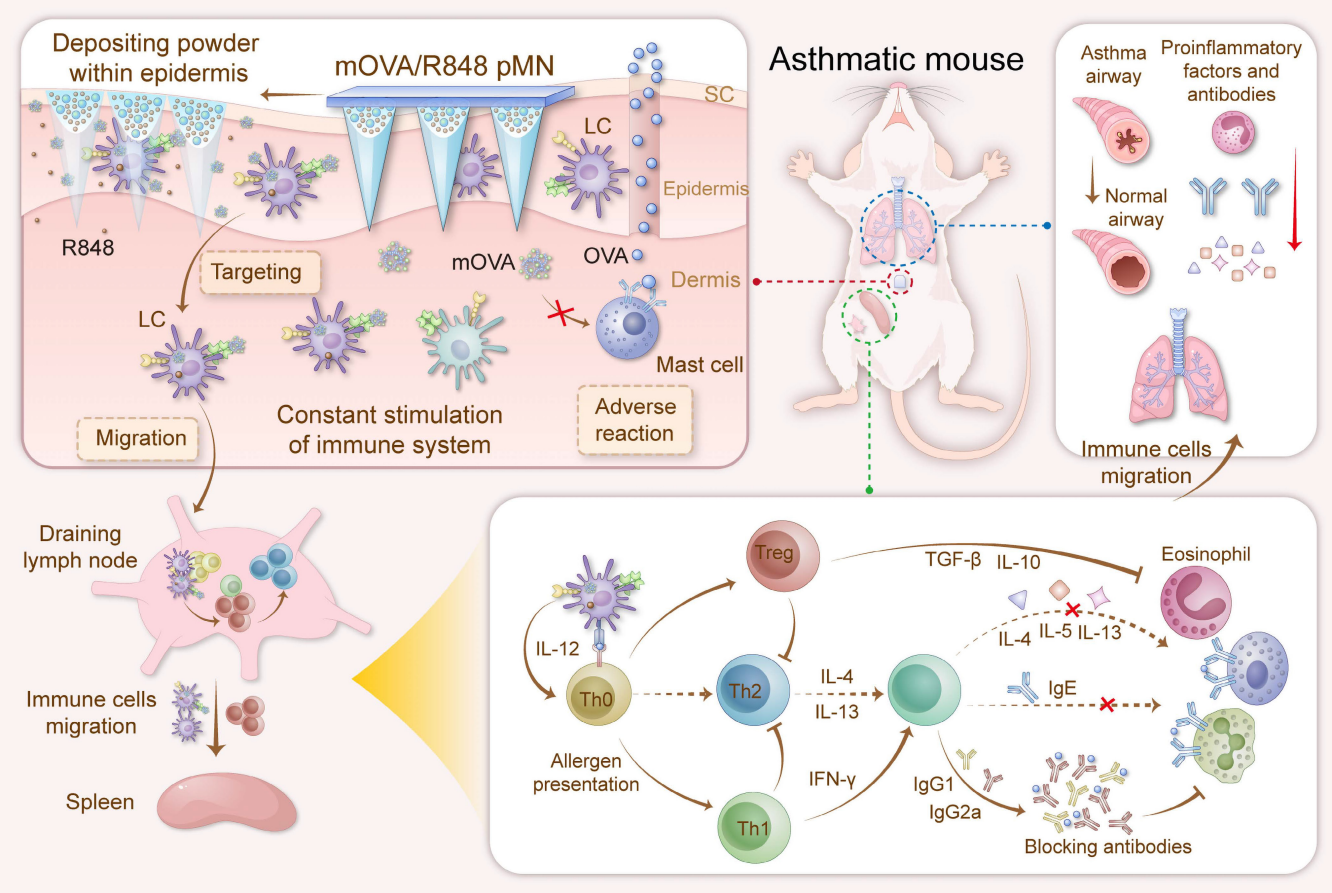
Optimized mOVA/R848 pMN arrays induce a protective immune response to alleviate allergic symptoms during allergen challenge. The powder-laden mOVA/R848 pMN arrays deposit powdery hypoallergens/immunomodulators within the epidermis, constantly targeting skin DCs and stimulating the immune system for days. Repeated treatment with mOVA/R848 pMN restores the immune response and effectively prevents allergic asthma progression during allergen challenge.

To circumvent the aforementioned obstacles and enhance the efficacy of AIT, we propose the use of powder-laden microneedles (pMNs) for the sustained epidermal delivery of powdery hypoallergens and immunomodulators (Figure 1). Specifically, targeted hypoallergenic derivatives, named mannan-ovalbumin (mOVA) conjugates, were synthesized. The pMNs were made of highly biocompatible silk fibroin (SF) with a cavity in the basal portion of each needle, and the cavities were filled with lyophilized mOVA and R848 powders (mOVA/R848 pMN). Once inserted into the skin, the mOVA/R848 pMN arrays deposited powder within the epidermis for an extended period, constantly targeting skin DCs and stimulating the immune system for days. Repeated treatment with mOVA/R848 pMN arrays suppressed Th2 immunity via Treg/Th1 bias with anti-inflammatory cytokines and neutralizing antibodies, thereby effectively alleviating allergic symptoms during allergen challenge. This work offers a perspective on improving cutaneous AIT.

## Results and Discussion

### Synthesis and characterization of mOVA conjugates

**Figure 2:**
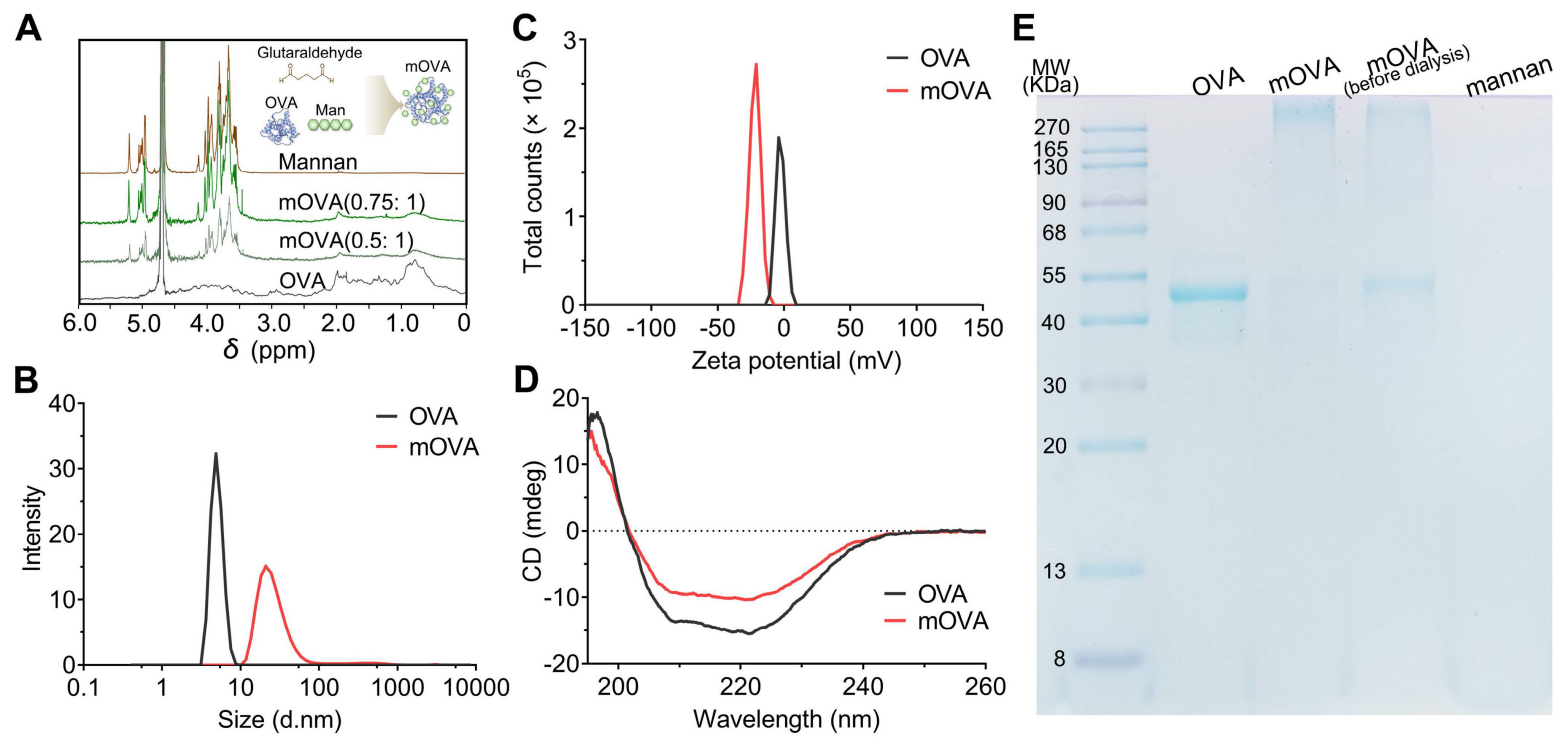
**Synthesis and characterization of mOVA conjugates. (A)** H-NMR spectra of OVA, mannan and mOVA conjugates at different ratios. Effect of mannan modification on **(B)** size distribution and **(C)** ζ-potential of mOVA conjugates. **(D)** CD spectra of OVA and mOVA conjugates. **(E)** SDS‒PAGE analysis of OVA, mOVA, mOVA (before dialysis) and mannan.

The conjugation of OVA allergen to mannan was performed with glutaraldehyde in a single-step process 26. Carbohydrate-modified allergens combine DC targeting with hypoallergenicity, thereby presenting the potential for low-dose AIT while concurrently minimizing the risk of adverse side effects. In contrast to the mannan oxidation process, the glutaraldehyde approach allows the preservation of mannose with its native geometry, which may be functionally important for receptor-mediated recognition. In this study, various mannan:OVA ratios were employed for conjugation, and the structural characterization of the mannan-OVA (mOVA) conjugates was conducted using H-NMR spectroscopy. After the conjugation process, specific saccharide signals were present in the mOVA conjugates but not in the OVA molecules (Figure 2A). The reduced translational diffusion in the mOVA conjugates indicated an increase in the average molecular size, suggesting the formation of supramolecular entities formed by the association between OVA and mannan (Figure S1) 27. The mOVA conjugates at a ratio of 0.75:1 presented the highest molecular weight and the highest carbohydrate content (42%). Increasing the mannan ratio to 1:1 did not increase the percentage of total carbohydrates or proteins (Table S1). Moreover, the alterations in size distribution and ζ-potential confirmed the formation of mOVA conjugates (Figure 2B-2C). The mOVA conjugates (0.75:1) had a diameter of 28.5 ± 2.6 nm and a highly negative ζ-potential of -21.0 ± 3.6 mV, whereas the highest percentage of OVA molecules measured 4.9 nm in diameter. The CD spectrum demonstrated minima at 208 and 222 nm and a maximum at 191 nm, which were indicative of an α-helical configuration (Figure 2D). However, these characteristic features were diminished in mOVA, suggesting that mOVA might possess a slightly less compact structure than native OVA does 28. Additionally, 44.3 kDa protein bands were observed for OVA and mOVA (before dialysis); for mOVA, the 44.3 kDa protein band was very faint. These results reflected the successful synthesis of mOVA after the polymerization and dialysis process (Figure 2E).

### Fabrication and characterization of powder-laden microneedle arrays

**Figure 3:**
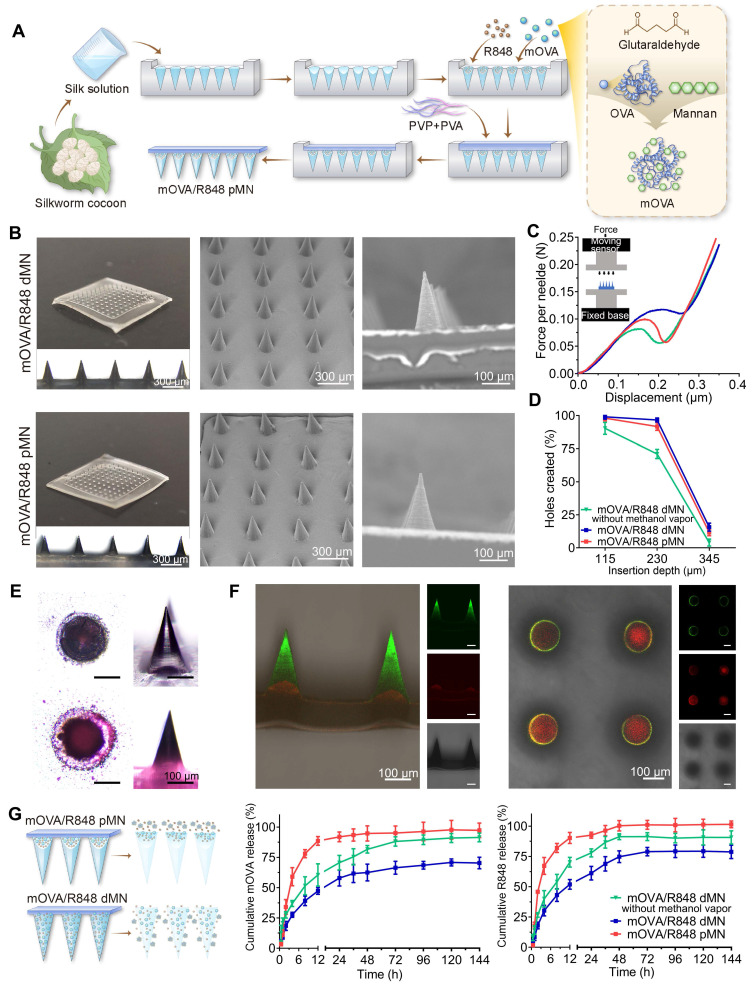
** Fabrication and characterization of powder-laden microneedle arrays. (A)** Fabrication of mOVA/R848 pMN arrays. **(B)** Representative microscopy and SEM images of mOVA/R848 dMN and mOVA/R848 pMN arrays. Scale bars = 100 μm and 300 μm. **(C)** Force‒displacement curves for mOVA/R848 dMN and mOVA/R848 pMN arrays. **(D)** Holes created on Parafilm. **(E)** Microscopy images of the pedestal and a complete needle body. **(F)** Representative CLSM images of vertical-sections and cross-sections of a powder-laden mOVA/R848 pMN, which displays a red cavity labelled with rhodamine B and a complete needle body stained with fluorescein sodium. Scale bars = 100 μm. **(G)** *In vitro* release behaviours of mOVA and R848 from mOVA/R848 pMN and mOVA/R848 dMN arrays. The data are presented as means ± SDs (*n* = 3).

Following the fabrication methods shown in Figure 3A and Figure S2, we prepared powder-laden microneedle (pMN) arrays, including OVA pMN, mOVA pMN, mOVA/R848 pMN, R848 pMN, and dissolving microneedle (dMN) arrays, named mOVA/R848 dMN. As shown in Figure 3B, each needle had a conical shape and was aligned on the backing. The powder-laden mOVA/R848 pMN arrays exhibited a height of 282 ± 3 μm, base width of 151 ± 8 μm, and needle pitch of 492 ± 2 μm, whereas the mOVA/R848 dMN arrays had a height of 276 ± 3 μm, base width of 152 ± 7 μm, and needle pitch of 499 ± 3 μm. The addition of powder to the cavity in the basal portion of each needle did not affect the surface or shape of the needle.

The mechanical strength and insertion ability were analysed to evaluate the skin-penetrating performance of the power-laden mOVA/R848 pMN arrays. According to the force‒displacement curve, each needle of mOVA/R848 pMN array endured forces of up to 0.10 ± 0.03 N before bending or breaking, indicating sufficient mechanical strength for manual skin penetration (Figure 3C) 29. Moreover, the compressive forces of the mOVA/R848 dMN arrays exceeded 0.12 ± 0.02 N per needle, which was significantly greater than that without methanol vapour annealing. The formation of cavities and the incorporation of powder compromised the mechanical integrity, resulting in a slightly reduced strength compared with that of mOVA/R848 dMN. When the fabricated mOVA/R848 pMN and mOVA/R848 dMN arrays were applied to Parafilm or excised skin, more than 95% of the needles created holes, suggesting that both arrays maintained adequate sharpness and stiffness. Histological analysis using haematoxylin and eosin (H&E) staining confirmed that the mOVA/R848 pMN arrays penetrated through the two topmost layers of the skin, enabling effective powder delivery to the epidermis and upper dermis (Figure 3D, Figure S3).

### Drug loading and release in powder-laden microneedle arrays

To visualize the powder distribution in the mOVA/R848 pMN arrays, rhodamine B was incorporated into the cavity as previously described, whereas fluorescein isothiocyanate was mixed with SF. As illustrated in Figure 3E, a cavity formed in the basal part of each needle as a result of condensation during polymerization, and the red rhodamine B powder was localized at the basal portion of conical needles 30. Bright red fluorescence was observed only within approximately 40 μm from the base, whereas green fluorescence signals extended to the full height of approximately 280 μm. A cross-sectional view revealed a core labelled red with rhodamine B surrounded by a shell stained green with fluorescein sodium dye, confirming that the cavity formed at the base (Figure 3F). Thus, the cavities could be filled directly with powdery allergen/adjuvant combinations through multiple rounds of centrifugation. This approach can be extended to all powdery bioactive substances because it does not interfere with drug properties such as solubility.

The fluorescence analysis revealed that the power-laden OVA pMN, mOVA pMN, mOVA/R848 pMN, and dissolving mOVA/R848 dMN arrays contained 27.1 ± 1.2 μg, 25.7 ± 2.8 μg, 25.4 ± 3.0 μg, and 25.3 ± 2.5 μg of OVA, respectively. HPLC revealed R848 contents of 2.3 ± 0.5 μg, 2.1 ± 0.3 μg and 2.8 ± 0.2 μg in the mOVA/R848 pMN, mOVA/R848 dMN and R848 pMN arrays, respectively (Table S2).

The release profiles of mOVA and R848 from mOVA/R848 pMN and mOVA/R848 dMN arrays were investigated *in vitro*. When mOVA/R848 pMN arrays were incubated in PBS, approximately 60% of the mOVA content and 67% of the R848 content were released within the first 4 h (Figure 3G). Here, the pedestals made of PVA and PVP disappeared within several seconds, so the drugs in the cavities were completely exposed to PBS, thereby promoting burst release of the encapsulated drug. The dissolving mOVA/R848 dMN arrays demonstrated sustained and stable release, with an accumulated release of approximately 70% of the mOVA content and approximately 79% of the R848 content. The dissolving MNs made from biodegradable SF exhibited drug release through mechanisms of swelling, erosion, and diffusion of the needle tips, which generally followed first-order release kinetics, with a decreased release rate over time 31. The release rate decreased as the β-sheet content in SF increased, which differed from the release rate for the mOVA/R848 dMN arrays without methanol vapour 32.

### *In vitro* allergen internalization and cell maturation

**Figure 4:**
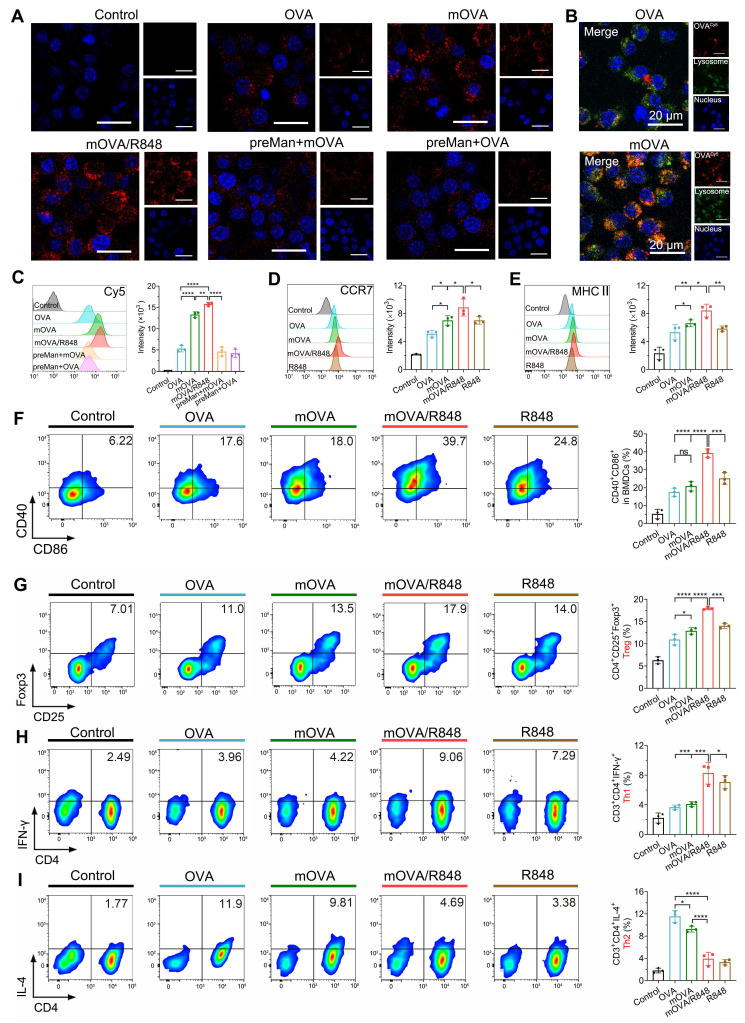
***In vitro* cell uptake, maturation and differentiation. (A)** CLSM images of DC2.4 cells pretreated with or without mannan and then incubated with OVA, mOVA, or mOVA for 4 h. **(B)** Representative CLSM images of OVA and mOVA colocalization in DC2.4 cells. The nuclei were stained with Hoechst 33258 (blue), and the lysosomes were stained with LysoTracker Green. OVA or mOVA was labelled with Cy5 (red). Scale bars = 20 μm. **(C)** Flow cytometry analysis of BMDCs pretreated with or without mannan and then incubated with OVA, mOVA, or mOVA for 4 h. BMDCs were labelled with anti-CD11c. **(D)** Expression of CCR7 on BMDCs. **(E)** Expression of MHC II on BMDCs. **(F)** Representative flow cytometry images and statistical data showing the expression of the CD40^+^CD86^+^ costimulatory molecules *in vitro*. The cells were stained with antibodies against CD11c. **(G)** Flow cytometry analysis of CD4^+^CD25^+^Foxp3^+^ Tregs induced by allergens/adjuvants. **(H)** Flow cytometry analysis of CD3^+^CD4^+^IFN-γ^+^ Th1 cells induced by allergens/adjuvants. **(I)** Flow cytometry analysis of CD3^+^CD4^+^IL-4^+^ Th2 cells induced by allergens/adjuvants. The data are presented as means ± SDs (*n* = 3). *P < 0.05, **P < 0.01, ***P < 0.001, and ****P < 0.0001.

The efficient uptake of allergens by dendritic cells (DCs) represents the critical step in initiating a specific immune response. mOVA and OVA were labelled with Cy5 to investigate their uptake and intracellular trafficking via CLSM (Figure 4A). Compared with the OVA group, the mOVA and mOVA/R848 groups presented much stronger fluorescence intensities in DC2.4 cells, indicating enhanced cellular uptake after mannan decoration. The increased cellular uptake of allergens/adjuvants by DCs was highly favourable for inducing effective immune responses. Moreover, allergen uptake by BMDCs was quantitatively evaluated using flow cytometry (Figure 4C). The fluorescence of mOVA with or without R848 was 2.97-fold and 2.51-fold greater than that treated with OVA alone, whereas blocking with mannan significantly decreased the uptake of glycated mOVA. These data clearly demonstrated that the presence of a targeting moiety benefited endocytosis. The mannose receptor (MR, CD206) and dendritic cell-specific intercellular adhesion molecule 3-grabbing nonintegrin (DC-SIGN; CD209) receptor are the main C-type lectin receptors (CLRs) on the surface of DCs that preferentially recognize mannose residues 5. In addition, MR-mediated endocytosis enables the quick and successive accumulation of antigens for subsequent major histocompatibility complex (MHC) presentation through constitutive recycling 6. To determine the intracellular localization of allergens, endosomes and lysosomes were stained with LysoTracker Green. CLSM images revealed that mOVA (red) colocalized with lysosomes (green) after internalization, suggesting that mOVA was generally processed in endosomal and lysosomal compartments for MHC II presentation, which subsequently generated CD4^+^ T cell responses (Figure 4B).

Next, we examined the ability of mOVA and R848 to regulate surface costimulatory molecules on BMDCs. As shown in Figure 4D-4F, all the stimulated BMDCs presented increased C-C chemokine receptor 7 (CCR7) expression. Notably, mOVA/R848-treated BMDCs presented the highest level of CCR7 expression, with increases of 1.74-fold, 1.27-fold, and 1.26-fold compared with OVA, mOVA, and R848 treatments, respectively. It needs to mention that CCR7-triggered skin DC migration towards dLNs is critical for the initiation of protective immunity 33. Similarly, the OVA, mOVA, mOVA/R848 and R848 groups presented elevated expression of surface MHC II, CD40 and CD86, suggesting the effectiveness of OVA, mOVA, mOVA/R848 and R848 in stimulating BMDCs. Importantly, mOVA/R848 increased the percentage of BMDCs that expressed CD40^+^CD86^+^ by 2.24-fold compared to OVA. These results indicated that both OVA and mOVA could stimulate BMDCs, and the superior DC activation ability of mOVA/R848 could be attributed to the agonistic effect of R848.

### *In vitro* T cell differentiation

Following allergen capture, immature DCs can be stimulated into mature DCs, accompanied by allergen presentation on their surface to activate T cells and induce subsequent immune responses. Thus, pretreated BMDCs were cocultured with lymphocytes from sensitized mice to examine T cell polarization 34. After 72 h of incubation, more CD4^+^CD25^+^Foxp3^+^ regulatory T cells (Tregs) were found in BMDCs treated with OVA, mOVA, mOVA/R848, or R848 than in control BMDCs. We observed that mOVA induced more Tregs than OVA did, and the mOVA/R848 group presented the highest percentage of Tregs (17.98%) (Figure 4G). The mannan-modified OVA targeted DCs via MR and DC-SIGN, enhancing allergen uptake and promoting the generation of functional allergen-specific Foxp3^+^ Tregs. Moreover, the secretion of transforming growth factor-β (TGF-β) and interleukin-10 (IL-10) strongly increased after mOVA treatment (Figure S4). Interestingly, mOVA promoted T cells to produce higher levels of IL-10 than did OVA, whereas R848 generated TGF-β-producing Tregs. The major function of Tregs and regulatory cytokines is to promote immune tolerance by various mechanisms 35.

The proportions of T helper 1 (Th1) and T helper 2 (Th2) cells were evaluated. Compared with OVA or mOVA treatment, BMDCs with mOVA/R848 enhanced Th1 polarization by 2.26-fold and 2.02-fold, respectively (Figure 4H). Conversely, Th2 proliferation was notably suppressed in the mOVA/R848 group, with a Th2 proportion of nearly 3.90% (Figure 4I). Consistent with the Th1/Th2 proportions, mOVA/R848 increased the secretion of interleukin-12 (IL-12) and interferon-γ (IFN-γ) but inhibited the production of Th2 cytokines (IL-4, IL-5 and IL-13) (Figure S5). A predominant Th2 response and the secretion of relevant cytokines into the local milieu are major contributors to allergic disease. R848 may bias the immune response towards Th1 and Treg types, which downregulate allergic Th2 inflammation, in accordance with T cell subset changes during the early stage of AIT 36, 37. The combination of mOVA and R848 not only inhibited the polarization of Th2 cells but also promoted the differentiation of Treg/Th1 cells, thus exerting stronger protective effects.

### Kinetics of allergen capture and their migration to draining lymph nodes

**Figure 5:**
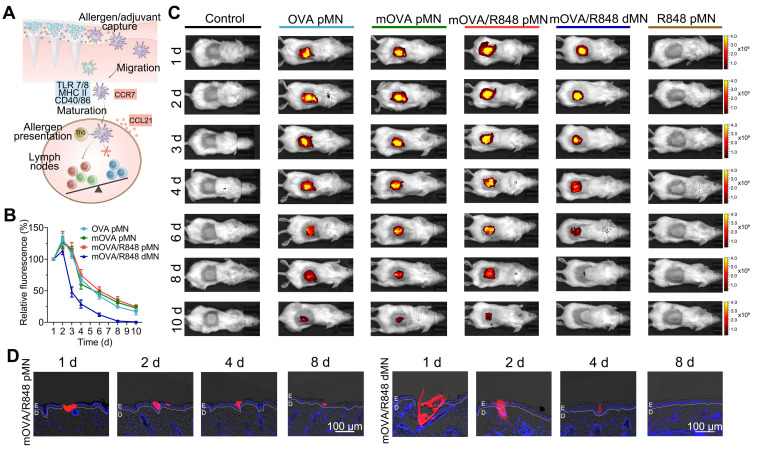
***In vivo* skin retention. (A)** Schematic illustration showing mOVA/R848 pMN application and skin DC migration. **(B)** Time-dependent decrease in the fluorescence signal of Cy5 after MN insertion. The fluorescence intensity was normalized to the maximal intensity collected on day 1. The data are presented as means ± SDs (n = 3). **(C)** Representative images of BALB/c mice acquired by the IVIS system at the indicated time points after the insertion of OVA pMN, mOVA pMN, mOVA/R848 pMN, mOVA/R848 dMN or R848 pMN arrays (containing 25 μg of OVA^Cy5^ and 2.5 μg of R848). **(D)** Fluorescence images of skin sections at days 1, 2, 4, and 8. E: epidermis, D: dermis.

Because skin-mediated AIT capitalizes on the ability of skin-resident DCs, such as LCs and dermal DCs, to capture allergens/adjuvants and migrate towards dLNs to induce allergen-specific immune responses, we evaluated the migration kinetics of skin DCs after MN application (Figure 5A) 38. To assess *in vivo* accumulation behaviour, we employed an *in vivo* imaging system to detect fluorescence signals at the insertion site. For the arrays loaded with allergen/adjuvant powders, including the OVA pMN, mOVA pMN and mOVA/R848 pMN arrays, the average fluorescence intensity of Cy5 gradually decreased, remaining detectable on day 10 postinsertion. In contrast, the Cy5 signal in mOVA/R848 dMN group became nearly undetectable after 6 d (Figure 5B-5C). After removing the mOVA/R848 pMN arrays 1 h after application, we observed that their shafts had separated out. Within the skin, red fluorescence from the powder was observed in the epidermis, which was highly significant at days 1 and 2 but declined over days 4 to 8 until all of the powder was ingested (Figure 5D). These powdery allergens may have drained into the epidermis by sucking interstitial fluid from the skin and diffusing against the interstitial fluid influx. In contrast, the red fluorescence from the mOVA/R848 dMN arrays was located both in the skin epidermis and dermis, and it decreased significantly after 4 d. The persistent allergens within each skin microchannel may stimulate the immune system continuously for more than 10 d, similar to multiple immunizations, which are known to favour immune tolerance.

**Figure 6:**
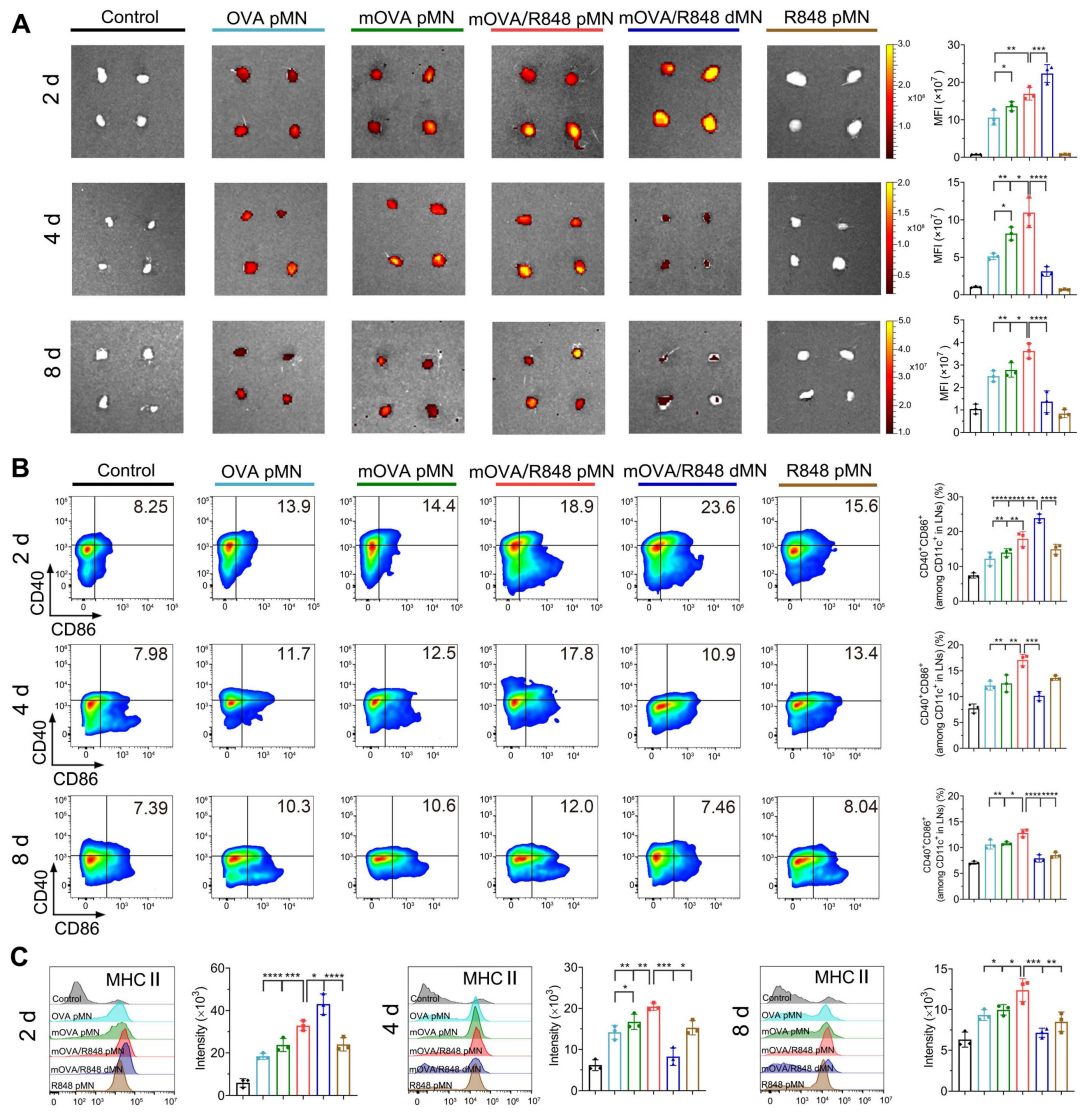
***In vivo* migration to draining lymph nodes. (A)** *Ex vivo* fluorescence images and quantitative analysis of the Cy5 channel in isolated dLNs at days 2, 4, and 8. **(B)** Representative flow cytometry images and statistical data showing the percentage of CD40^+^CD86^+^ on CD11c^+^ DCs in inguinal LNs at days 2, 4, and 8. **(C)** MHC II expression in dLNs gated on CD11c^+^ DCs at days 2, 4, and 8. The data are presented as means ± SDs (n = 3). *P < 0.05, **P < 0.01, ***P < 0.001, and ****P < 0.0001.

The dLN tissues from each group were collected, and their fluorescence signals were recorded. Images revealed the accumulation of OVA^Cy5^ or mOVA^Cy5^ in axillary and inguinal LNs on day 2, indicating successful trafficking from the active epidermis to skin dLNs (Figure 6A). The Cy5 fluorescence from the mOVA/R848 dMN group was greater than that from the OVA pMN, mOVA pMN and mOVA/R848 pMN arrays on day 2, which suggested that the dissolving mOVA/R848 dMN array efficiently delivered mOVA^Cy5^ after insertion, resulting in the highest accumulation in a short time. The allergens were continuously enriched in dLNs, but the fluorescence levels decreased rapidly thereafter. Among all, the mOVA/R848 pMN group presented the highest fluorescence on day 4, with 2.13-fold, 1.34-fold and 3.51-fold increases compared with those observed in the OVA pMN, mOVA pMN and mOVA/R848 dMN groups, respectively. During this period, powders in mOVA/R848 pMN were abundant within skin microchannels, facilitating ongoing trafficking to the dLNs. Compared with the OVA pMN group, the mOVA pMN group presented a 1.60-fold greater fluorescence intensity, highlighting the superior capability of mannan decoration. Importantly, the mOVA/R848 pMN group showed the highest Cy5 signals on day 4, verifying that the targeting moieties and immune adjuvants promoted draining. These signals were well maintained in the LNs even after 8 d in the groups that contained powdery allergens/adjuvants, namely, the OVA pMN, mOVA pMN and mOVA/R848 pMN groups.

Flow cytometry analysis was employed to characterize the phenotype of migrating DCs in dLNs, using CD40, CD86 and MHC II as mature cell surface markers. As shown in Figure 6B, all the loaded arrays triggered significant DC maturation. Notably, on day 2, compared with the OVA pMN, mOVA pMN and mOVA/R848 pMN groups, the mOVA/R848 dMN group exhibited the highest proportion of CD40**^+^** and CD86**^+^** double-positive cells. However, the mOVA/R848 pMN group presented the highest percentage of CD40^+^CD86^+^ DCs at days 4 and 8, with increases of 1.68-fold and 1.62-fold, respectively, compared with those of the mOVA/R848 dMN group. These findings provided further evidence that the mOVA/R848 pMN array maintained an obvious stimulatory effect on day 8, whereas that of mOVA/R848 dMN decreased rapidly over time. In addition, high levels of MHC II expression were maintained in the mOVA/R848 pMN group throughout this period, whereas the MHC II expression levels returned to baseline levels in the mOVA/R848 dMN group after 8 d (Figure 6C). These results highlighted the unique advantages of mOVA/R848 pMN for efficient lymph node draining and sustained immune induction.

### Preliminary safety evaluation of powder-laden microneedle arrays* in vitro* and* in vivo*

**Figure 7:**
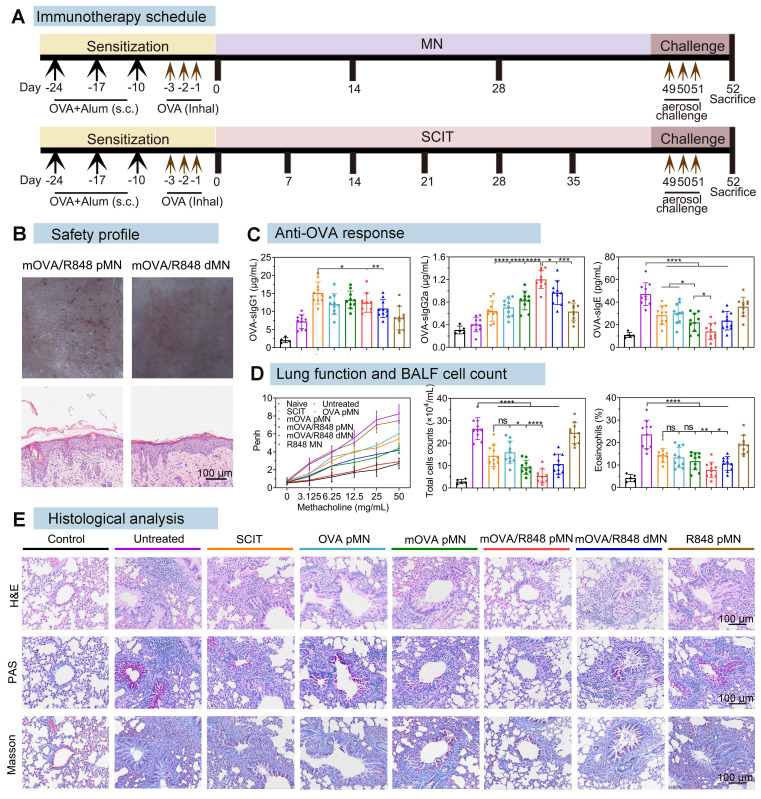
** mOVA/R848 pMN treatment alleviates airway hyperresponsiveness and lung inflammation in asthmatic mice. (A)** Schematic of anaphylaxis and therapeutic regimen. **(B)** Skin responses and H&E-stained sections after mOVA/R848 pMN and mOVA/R848 dMN treatment. Scale bars = 100 μm. **(C)** Quantification of OVA-specific IgE, OVA-specific IgG1, and OVA-specific IgG2a in the serum. **(D)** Penh values under increasing methacholine concentrations. Total cell counts in BALFs. Percentage of eosinophils in BALFs. The data are presented as means ± SDs (naive:* n* = 5; other: *n* = 10). *P < 0.05, **P < 0.01, ***P < 0.001, and ****P < 0.0001. **(E)** Representative H&E, PAS and Masson staining of lung sections. Scale bars = 100 μm.

The well-established OVA-induced murine model of allergic airway disease was used to investigate allergy pathogenesis and develop new treatments against allergies (Figure 7A). Prior to confirming the effectiveness of powder-loaded mOVA/R848 pMN arrays, their safety profiles were explored. When cocultured with the materials required for fabrication or with the prepared mOVA/R848 pMN arrays, the 3T3 cell morphology remained intact, and the cell viability was greater than 95% (Figure S6). After mOVA/R848 pMN application, there was no obvious local inflammation at the treated site, compared with the negative control group. Pathological analysis of the skin at the mOVA/R848 pMN application site revealed that the collagen fibres in the dermis were neatly arranged, and lymphocyte infiltration was observed. The epidermis of the mice treated with MN arrays was significantly thicker than that of the control group (Figure 7B, Figure S7).

### Alterations of immunoglobulin levels in serum

After intraperitoneal sensitization with OVA and Alum, followed by intranasal challenge with OVA, the model mice exhibited massive inflammatory cell infiltration and thickening of the airway epithelium, confirming that the allergic asthma model was successfully established (Figure S8). For MN-mediated delivery, the mice received three treatments with the OVA pMN, mOVA pMN, mOVA/R848 pMN, mOVA/R848 dMN or R848 pMN arrays. For SCIT group, the mice were administered OVA with Alum six times. Following three consecutive days of high-dose OVA to simulate allergen exposure, the levels of OVA-specific IgE, IgG1 and IgG2a (sIgE, sIgG1 and sIgG2a) were measured to assess therapeutic efficacy.

The generation of allergen-specific IgG with IgE inhibitory activity during repetitive allergen exposure contributes to long-term tolerance 39. As shown in Figure 7C, all groups receiving AIT presented significantly higher sIgG1 levels than the untreated group did, validating the activation of protective effects. The SCIT group demonstrated higher sIgG1 levels than the mOVA/R848 pMN and mOVA/R848 dMN groups. sIgG1 serves as a blocking antibody that can compete with IgE for allergens, thus preventing the formation of allergen-IgE complexes from binding to FcεRI on effector cells 40. Compared with those in SCIT group, there were higher levels of the sIgG2a subtype in the mOVA/R848 pMN and mOVA/R848 dMN groups. The IgG1/IgG2a ratio decreased significantly after treatment with R848, verifying the shift of immune response from a Th2 to a Th1 pattern due to the presence of R848 (Figure S9). Several reports have indicated that R848 activates Th1-related lymphocytes, whereas SCIT with Alum results in Th2-skewed immunity with a stronger IgG1 response 41. Moreover, mannan decoration increased the production of sIgG2a subclass antibodies.

Allergy is an IgE-dependent hypersensitivity in atopic individuals because IgE triggers the immediate release of inflammatory mediators from mast cells and basophils by allergen recognition and receptor cross-linking 2. sIgE production was consistently inhibited in all treated mice following the discontinuation of AIT and allergen challenge. Comparable levels of sIgE between SCIT and OVA pMN groups clearly suggested that the powder-loaded OVA pMN arrays could reduce the number of treatments by half while maintaining similar therapeutic efficacy. Importantly, the serum sIgE levels in the mOVA/R848 pMN group were significantly lower than those in the OVA pMN, mOVA pMN, and mOVA/R848 dMN groups, indicating that mOVA/R848 pMN reduced the therapeutic frequency and stimulated a superior anti-OVA response in asthmatic mice.

### Alleviation of airway hyperresponsiveness and lung inflammation

To assess lung function, the enhanced pause (Penh) in response to methacholine in allergic asthma model mice was measured by whole-body plethysmography on the day following three consecutive days of challenge 42. With a concentration of 50 mg/mL methacholine, the mean Penh values decreased by 1.51-fold in SCIT, 1.36-fold in OVA pMN, 1.88-fold in mOVA pMN, and 1.97-fold in mOVA/R848 dMN, compared with those in the untreated group (mean Penh 8.23) (Figure 7D). The Penh values in mOVA/R848 pMN group remained comparable to those in the naive group, confirming that treatment with mOVA/R848 pMN significantly alleviated respiratory symptoms in asthmatic mice during methacholine stimulation.

Successful AIT induces allergen-specific peripheral tolerance, which is characterized primarily by the generation of allergen-specific Tregs and a reduction in Th2 cells, both of which are crucial for alleviating lung inflammation 39. In terms of the relative cytokine levels in bronchoalveolar lavage fluids (BALFs), significant increases in Th2 cytokines (IL-4, IL-5 and IL-13) were observed in untreated mice compared with those in naive mice (Figure S10). Compared with SCIT, the addition of R848 as an adjuvant with allergens (mOVA/R848 pMN and mOVA/R848 dMN) considerably suppressed the expression of IL-4, IL-5 and IL-13 in BALFs after three treatments. Theoretically, Th2 cytokines are critical mediators for allergic inflammation. It has been reported that IL-4 activates various immune cells and increases IgE levels and that IL-5 represents the most important biological factor responsible for eosinophil recruitment. IL-13 is involved in bronchial hyperactivity and aggravates airway obstruction 43. In contrast to Th2 cytokines, the anti-inflammatory cytokines TGF-β and IL-10 exert inhibitory effects on T cell differentiation to suppress the allergic response. A trend towards higher TGF-β and IL-10 expression was observed in MN-treated mice than in untreated mice (Figure S11). Specifically, the mean concentration of TGF-β from mOVA/R848 pMN group was 208.04 pg/mL, which was 3.10-fold greater than that in untreated group, 1.69-fold greater than that in OVA pMN group, and 1.57-fold greater than that in mOVA pMN group, confirming the combination of mannan decoration and R848. In addition, the IL-10 concentration was greater after AIT, but no significant difference was observed among the SCIT, OVA pMN and mOVA pMN groups. IL-10 is an essential suppressive factor during allergic responses in the lung, whereas R848 generates TGF-β-producing Tregs that maintain pulmonary homeostasis by inhibiting both Th2 and Th1 responses and has been implicated in the control of airway inflammation 44. Naive group presented low quantities of both pro- and anti-inflammatory cytokines.

To further substantiate the protective effect of mOVA/R848 pMN, the cell counts in BALFs were determined following plethysmography (Figure 7D). The total cell counts (including lymphocytes, eosinophils, macrophages, etc.) in untreated and R848 pMN groups were significantly greater than those in SCIT, OVA pMN, mOVA pMN and mOVA/R848 dMN groups. Compared with those in SCIT group, similar results were observed in OVA pMN group with three treatments, suggesting that MN could be considered an efficacious and safe alternative to traditional SCIT. Notably, the percentage of total cells was 4.94-fold lower in mOVA/R848 pMN group than in untreated group. This trend remained unaltered for percentages of eosinophils. The mOVA/R848 pMN treatment reduced the infiltration of eosinophils and lymphocytes in the airway, thus normalizing the total cell counts. We also evaluated the level of reactive oxygen species (ROS) in lung tissues by DHE staining. There was obvious red fluorescence of ROS in untreated group, but the red fluorescence decreased substantially after mOVA/R848 pMN treatment (Figure S12). Excessive ROS production induces airway inflammation, tissue injury and remodelling 45. These results revealed that the localized immune system was able to cope with lung inflammation.

Consistent with these findings, analysis of lung tissue sections provided further insights (Figure 7E). H&E staining revealed significant inflammatory cell infiltration in both the peribronchial and alveolar regions of asthmatic lungs in untreated mice. Conversely, the SCIT, OVA pMN, mOVA pMN, and mOVA/R848 dMN groups all presented reduced inflammatory cell infiltration and decreased airway epithelial thickness. The mice treated with mOVA/R848 pMN presented clear alveolar spaces and normal bronchial walls, which appeared histologically normal. Additionally, mOVA/R848 pMN treatment prevented the hyperplasia of PAS-positive cells, which are mucus-producing goblet cells, while varying degrees of mucus production were observed in the other groups. Mucus hyperplasia is a hallmark of airway inflammation, wherein the secretion of mucus results in airway obstruction, which ultimately contributes to difficulty breathing and shortness of breath 46. Masson staining revealed that collagen deposition around the bronchioles was significantly reduced in the mOVA/R848 pMN group, indicating improved flexibility of the lung bronchioles. Overall, these results suggested that mOVA/R848 pMN-mediated AIT might be a promising therapy for alleviating allergen-induced AHR, inflammatory cell recruitment and histological changes in asthmatic models.

### Regulation of systemic immune response

**Figure 8:**
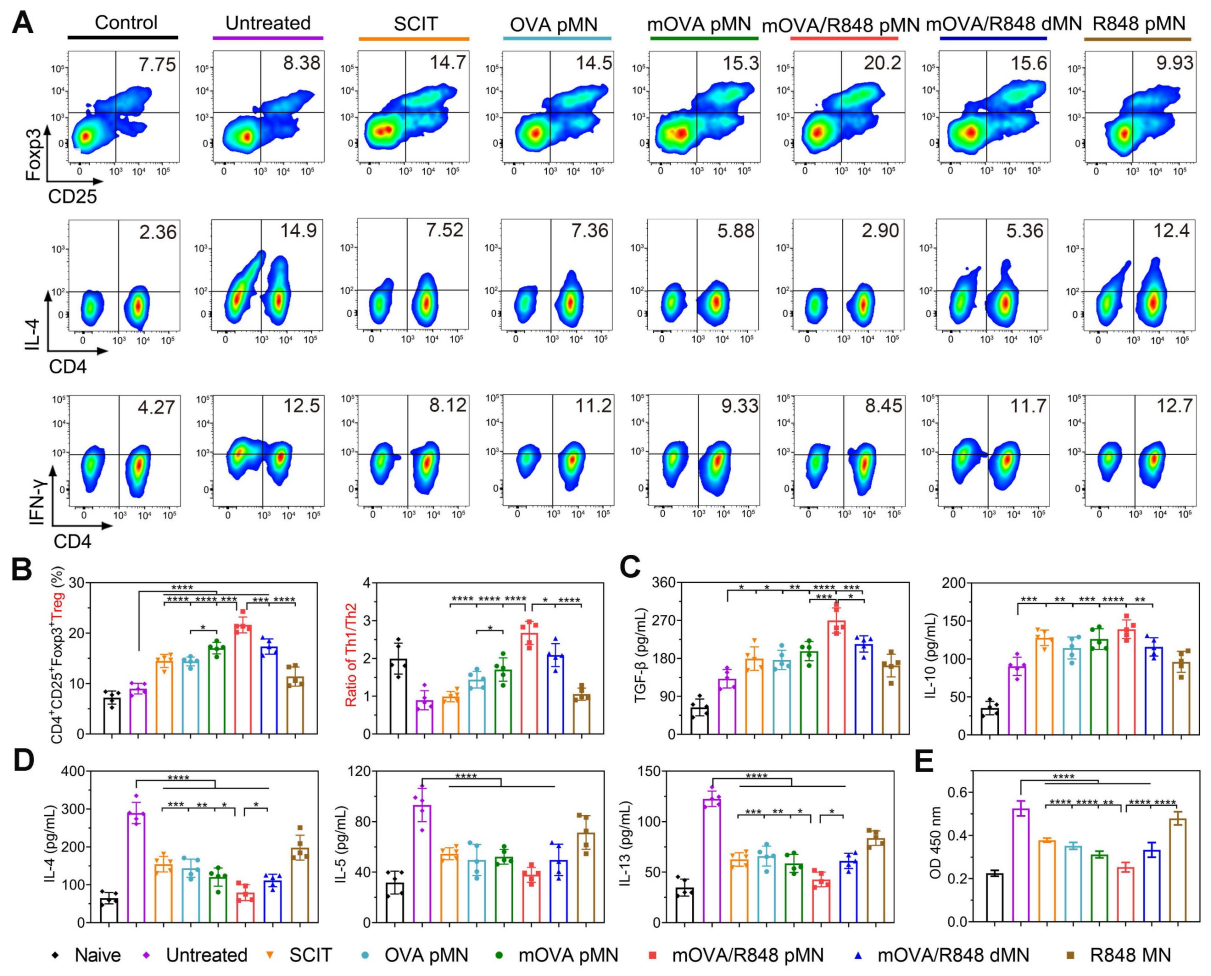
** mOVA/R848 pMN treatment regulates the systemic immune response.** Representative flow cytometry images showing **(A)** CD4^+^CD25^+^Foxp3^+^ Tregs, CD3^+^CD4^+^IL-4^+^ Th2 cells, and CD3^+^CD4^+^IFN-γ^+^ Th1 cells in the splenocyte population. **(B)** Proportion of Tregs and the Th1/Th2 ratio in the splenocyte population. **(C)-(D)** OVA-specific cytokine production in splenocytes.** (E)** OVA-specific proliferation of splenocytes. The data are presented as means ± SDs (*n* = 5). *P < 0.05, **P < 0.01, ***P < 0.001, and ****P < 0.0001.

To explore systemic immune responses, the cell differentiation and cytokine secretion by splenocytes were evaluated after *ex vivo* restimulation with OVA. The repeated mOVA/R848 pMN treatment induced the highest percentage of Tregs, with increases of 2.41-, 1.49-, 1.50-, 1.27-, and 1.25-fold compared with those in the untreated, SCIT, OVA pMN, mOVA pMN and mOVA/R848 dMN groups, respectively. The percentages of Tregs in the mOVA pMN, mOVA/R848 pMN and mOVA/R848 dMN groups were significantly greater than those in the SCIT group, suggesting that the designed MN system was a more effective alternative to SCIT. The differentiation of Tregs induces immune tolerance and alleviates autoimmune damage. Conversely, the proliferation of Th2 cells in treated mice was considerably suppressed. There was no significant difference in the Th2 proportion between mOVA/R848 pMN group and naive group, indicating the protective effect of mOVA/R848 pMN following discontinuation. Similarly, the proliferation of Th1 cells also partially decreased during allergen challenge (Figure 8A-8B, Figure S13). Here, splenocytes restimulated with OVA not only induced Tregs, which played a pivotal role in regulating allergic disease, but also inhibited the excessive proliferation of both Th1 and Th2 cells. Compared with SCIT and untreated groups, mOVA/R848 pMN treatment elicited Th1 skewing, with increased Th1/Th2 ratios and a decreased proportion of Th2 cells, suggesting that R848 application was beneficial for maintaining the Th1/Th2 balance.

Cytokine analysis revealed that the TGF-β level was greater in mOVA/R848 pMN group than in the other groups. Similarly, a trend towards higher IL-10 expression was observed in treated mice than in untreated mice (Figure 8C). Both Tregs and anti-inflammatory cytokines restored immune tolerance during allergen challenge. In contrast, the excretion of Th2 cytokines in mOVA/R848 pMN group was low and similar to that in naive group, suggesting the activation of the systemic immune response to cope with allergic inflammation (Figure 8D). Compared with untreated group, mOVA/R848 pMN treatment elicited a Th1-skewed response, with increased IFN-γ/IL-4, IFN-γ/IL-5, and IFN-γ/IL-13 ratios (Figure S14-S15). The allergen-specific shift of Th2- to Th1-type cytokines suppresses IgE-mediated allergic responses 47. Finally, splenocyte proliferation following OVA restimulation was evaluated (Figure 8E). There were significant immune responses in untreated group, whereas all the treated groups exhibited less proliferation, which suggested that the effective AIT decreased T cell proliferation. These results were in good agreement with local allergic inflammation. Overall, these data verified that mOVA/R848 pMN treatment shifted the Th2-driven immune landscape towards the Treg/Th1-cell-like phenotype in allergic mice and reduced both local and systemic allergic responses.

## Conclusions

To date, AIT represents the only curative treatment for IgE-mediated allergic disease. AIT not only controls allergy symptoms during allergen exposure but also affects the natural course of allergic disorders. Owing to the shortcomings of AIT in terms of efficacy and safety, the discovery of novel, safe and effective formulations for respiratory or food allergies has attracted considerable scientific interest. Emerging approaches emphasize self-administration modes that enable efficient allergen transfer and promote higher safety requirements. In this study, we proposed the use of powder-laden mOVA/R848 pMN, which consisted of the targeted hypoallergen mOVA and the immunomodulator R848 for sustained epidermal delivery. The deposited mOVA and R848 powders improved skin DC uptake and provided constant stimulation of the immune system after skin application. On the basis of these features, the mOVA/R848 pMN arrays increased the induction of protective immune mechanisms to suppress Th2 immunity, including Treg/Th1 bias, as well as the production of anti-inflammatory cytokines and neutralizing antibodies in an asthmatic mouse model. Furthermore, the combined administration of allergens and immunomodulators significantly increased the induction efficiency. Compared with traditional SCIT, a total dose of only 75 μg of OVA over three treatments was sufficient to restore immune balance in asthmatic mice and alleviate allergic symptoms during allergen exposure, highlighting the dose-sparing and frequency-sparing potential of the optimal mOVA/R848 pMN arrays.

The use of powder-loaded MNs can be extended to all antigen/adjuvant combinations without loss of antigenicity, as there is no interference with solubility during MN fabrication. This system is also applicable to autoimmune diseases, such as type 1 diabetes, rheumatoid arthritis, and psoriasis, and its direct encapsulation of drug powders greatly improves the loading capacity 48. However, MN arrays should be rationally designed for application purposes, considering their structural parameters, such as shape and size, which influence their mechanics and functions 49. Therefore, further investigations involving enlarged and prolonged MN arrays should be developed for human use owing to the much thicker skin of humans than that of mice. In addition, optimal variables such as allergen dosage, treatment duration and patient compliance may improve the desensitization effect, which would be necessary in humans for the transition to clinical applications. Overall, the mOVA/R848 pMN arrays developed in the present study yielded safer and more efficacious outcomes than conventional SCIT did, suggesting a new therapeutic strategy for allergy treatment with significant improvements in reliability and convenience.

## Methods and Materials

### Materials

Mannan from *S. cerevisiae* (average molecular weight of 46 kDa), ovalbumin (OVA, grade V) (molecular weight of 44.3 kDa), glutaraldehyde, concanavalin A (Con A) and methacholine chloride were purchased from Sigma‒Aldrich (USA). Polyvinyl pyrrolidone (PVP K90) and polyvinyl alcohol (PVA 1788) were purchased from Aladdin Reagent (Shanghai, China). Alhydrogel® adjuvant was purchased from InvivoGen (France). Trypsin-EDTA, DMEM, RPMI 1640 medium and fetal bovine serum (FBS) were obtained from Thermo Fisher Scientific (USA). Bradford protein assay kit, phosphate buffer solution (PBS), RBC lysis buffer, prestained protein marker (8-270 kDa), 5×loading buffer, 4′,6-diamidino-2-phenylindole (DAPI), LysoTracker Green and dihydroethidium (DHE) were obtained from Servicebio (Wuhan, China). Cyanine5 N-hydroxysuccinimide ester (Cy5-NHS) was obtained from Meilunbio (Dalian, China). Mouse spleen lymphocyte separation kit was purchased from Haoyang Biological Manufacture Co., Ltd. (Tianjin, China). The Cytofix/Cytoperm Fixation/Permeabilization Solution Kit, Transcription Factor Buffer Set and Leukocyte Activation Cocktail were purchased from BD Bioscience (USA). The antibodies used in this study were purchased from Universal Biotech Co., Ltd. (Shanghai, China) and are listed in Table S3. The ELISA kits used in this study were purchased from Yifeixue Biotechnology Co., Ltd. (Nanjing, China) and are listed in Table S4. The polydimethylsiloxane (PDMS) moulds consisted of a total of 10×10 needles, with both 300 µm in height and 160 µm in base length.

### Allergen conjugation with mannan from *S. cerevisiae*

The model allergen OVA was polymerized and conjugated with mannan (mOVA) in a single-step process 26. Mannan was mixed with 10 mg/mL OVA at an equal volume, and then conjugated with glutaraldehyde (final concentration of 25 mM) to obtain mOVA conjugates. The mixture was made at different mannan:OVA ratios (0.5:1; 0.75:1; 1:1) in PBS, and the reaction was performed for 6 h at 4 °C under continuous stirring, and then stopped with glycine (1.25 M). Free OVA and mannan molecules were removed by dialysis (100 kDa) with distilled water, after which the mOVA conjugate was further lyophilized until use.

### Characterization of mOVA conjugates

The total protein and carbohydrate contents of the mOVA conjugates were measured with the Bradford assay and the phenol‒sulfuric acid assay, respectively 50, 51. An OVA standard was produced, and linear regression was used to fit the standard curve. The samples were mixed with Bradford solution and incubated at 37 °C for 10 min. Absorbance was measured at 595 nm using a microplate reader (Varioskan LUX, Thermo Scientific, USA). A phenol‒sulfuric acid assay was used to measure the mannan concentration. Mannan was gradient diluted and used to make a standard curve. Then, 5% (w/v) phenol was mixed into the samples, followed by H_2_SO_4_ and mixing. The mixtures were incubated for 40 min at room temperature, and their absorbance was measured at 490 nm.

Sodium dodecyl sulfate‒polyacrylamide gel electrophoresis (SDS‒PAGE) was used to investigate the formation of mOVA conjugate. Every sample was mixed with 5×loading buffer and heated at 95 °C for 5 min. These samples were assessed by 10% SDS‒PAGE at 200 V for 30 min and stained with Coomassie blue R250.

Additionally, the particle size distribution and zeta potential of the mOVA conjugates were determined by dynamic light scattering (DLS, Zetasizer Nano ZS90, Malvern, UK). Circular dichroism spectroscopy (CD, J-1500, JASCO, Japan) was used to characterize the secondary structure of the mOVA conjugate. Nuclear magnetic resonance spectroscopy (NMR) was performed using Bruker Ascend TM 600 MHz (Bruker, Germany), including standard 1H-NMR and diffusion ordered spectroscopy (DOSY) experiments. For 2D-DOSY experiments, all samples were prepared at an OVA concentration of 400 μM with 75 mM PBS in 100% D_2_O 52. The experiments were carried out by recording 64-128 scans for each gradient step, a linear gradient of 16 steps between 2% and 95%, a diffusion time (big delta) between 0.2-0.4 s, and a length of the diffusion encoding gradient pulses (little delta) between 2-4 ms.

### Fabrication of powder-laden microneedle arrays

Silk fibroin (SF) was obtained according to a previously described method 53. Specifically, *Bombyx mori* silkworm cocoons were boiled in a Na_2_CO_3_ aqueous solution (0.02 M) and rinsed with excessive deionized water. Afterwards, the obtained glue-like sericin protein was dissolved in LiBr solution (9 M) at 60 °C for 4 h and dialyzed with deionized water for 48 h. Then, the SF solution was further lyophilized until use.

pMN arrays were fabricated through micromoulding technology, as shown in Figure 3A. An 8% SF solution in deionized water was added to the PDMS mould and centrifuged for 5 min at 3000 rpm/min twice to ensure that each cavity of the mould was evenly filled with SF solution and that no bubbles formed. Excess solution was removed from the mould gently and flatly using a cotton swab. The mould was then placed in a negative pressure container overnight, forming a cavity approximately 30 μm in depth in the upper portion and a sharp tip in the lower part of each needle. The powdery mOVA was mixed with R848 at a ratio of OVA/R848 equivalent to 10:1 before being added into the cavity. The mould was centrifuged for 5 min at 3000 rpm/min twice until all the cavities were fully laden 30.

Afterwards, the pedestal solution was prepared by adding 0.11 g of PVA 1788 and 0.10 g of PVP K90 to 1 mL of ethanol solution (50% v/v), and left to swell overnight. This solution was cast onto the surface of the PDMS mould, followed by centrifugation at 3000 rpm/min for 10 min and left overnight. Once dried, methanol vapour annealing was performed to improve the mechanical strength of the MN tips. The mould was placed in a desiccator containing 100 mL of methanol and set under vacuum for 1 h. After complete drying, the inner arrays were peeled off with transparent adhesive tape. As a control, mOVA and R848 were dissolved in SF solution, and dMN arrays named mOVA/R848 dMN were prepared (Figure S2).

### Characterization of powder-laden microneedle arrays

The prepared mOVA/R848 pMN or mOVA/R848 dMN arrays were inspected under light microscopy (X53, Olympus Corporation, Japan) to assess their shape and sharpness. The dimensions, including the height, width of base and needle pitch, were analysed with ImageJ. For high-resolution imaging, the arrays were coated with gold with an ion sputter coater (ISC150, SuPro Instruments, China), and images were recorded by scanning electron microscopy (SEM, Phenom Pure, Phenom Scientific Instrument, China) at 5 kV.

The mechanical properties were measured by a texture analyser (Universal TA, shtengba, China) following a previously described method 54. After being fixed to the text plate with tips facing up, the arrays were approached by the sensor probes at a speed of 0.05 mm/s until a displacement of 300 μm was reached. The axial force (N) was recorded as a function of displacement.

To evaluate the insertion capability *in vitro* and *in vivo*, the arrays were inserted into Parafilm or mouse skin, followed by removal of the residual patches. As an alternative to skin, Parafilm was folded to obtain an eight-layer film (approximately 1 mm thickness), and a poly(urethane) film was used as received 55. The probe of the texture analyser was lowered onto Parafilm or skin until the required force was exerted. Forces were held for 5 min at 10 N. After insertion, Parafilm was unfolded, and the number of holes created in each layer was detected under a digital microscope with two polarizer filters. To evaluate the skin penetration capacity, the arrays were applied to the dorsal skin of BALB/c mice with a self-made applicator. The applied skin was imaged, and the penetration efficiency (PE) of each individual array was calculated by counting the number of holes on the skin.

### Drug loading and release in powder-laden microneedle arrays

To observe the power distribution in mOVA/R848 pMN arrays, rhodamine B was added to the cavity as mentioned above, whereas the SF solution was mixed with a small amount of fluorescein isothiocyanate. The resulting arrays were imaged by confocal laser scanning microscopy (CLSM, FV3000, Olympus Corporation, Japan).

To measure drug loading, mOVA was fluorescently labelled with Cy5 (mOVA^Cy5^) according to the manufacturer's instructions. In brief, the prepared arrays were dissolved in 2 mL of PBS to release all the encapsulated powders. The concentration of mOVA^Cy5^ was determined by a microplate reader with an excitation wavelength of 625 nm and an emission wavelength of 670 nm. The amount of R848 was analysed through high-performance liquid chromatography (HPLC, LC-2030C, Shimadzu, Japan) and UV absorption at 254 nm on a ShimNex CS C18, 5 μm, 4.6 × 250 mm column (Shimadzu, Japan).

To observe the *in vitro* release profiles of mOVA^Cy5^ and R848, the prepared arrays were immersed in 2 mL of PBS and incubated at 32 °C. At predetermined time points, 200 μL of the supernatant was collected, and the medium was replaced with the same volume of fresh PBS. The concentration of mOVA^Cy5^ was determined by a microplate reader, and the amount of R848 was analysed through HPLC as described above.

### Cells and animal models

Dendritic cell line DC2.4 was purchased from the Shanghai Institute of Biochemistry and Cell Biology, Chinese Academy of Sciences (Shanghai, China). The cells were cultured in RPMI 1640 supplemented with 10% heat-inactivated FBS and 1% penicillin‒streptomycin.

Bone marrow-derived dendritic cells (BMDCs) were generated from the bone marrow of male BALB/c mice as previously described for cell internalization and maturation assays 56. The cells were cultured in RPMI 1640 medium supplemented with 10% heat-inactivated FBS, 10 ng/mL IL-4 and 20 ng/mL GM-CSF, and the medium was replaced every 2 days. On day 5, the cells were obtained as immature BMDCs for further experiments.

Female BALB/c mice (5 weeks) were purchased from Cavens Laboratory Animal Ltd. (Changzhou, China). All animal experiments were approved by the Jiaxing University Ethics Committee (JUMC2023-086) and carried out in accordance with the National Institute of Health Guide for the Care and Use of Laboratory Animals. The BALB/c mice were sensitized with a homogenous suspension containing 20 μg of OVA and 2 mg of Alum by three intraperitoneal injections at one-week intervals. One week after sensitization, the mice were exposed to 1% OVA aerosol for 30 min per day for three consecutive days. The back skin of each mouse was shaved with electric clippers 24 h before application, and depilatory cream was applied.

### *In vitro* allergen internalization and cell maturation

Cy5-labelled OVA and mOVA were synthesized, and their uptake and intracellular trafficking were studied with CLSM and flow cytometry. DC2.4 cells were inoculated into confocal dishes at 1 × 10^5^ cells per well and incubated overnight. They were exposed to OVA^Cy5^, mOVA^Cy5^ or mOVA^Cy5^/R848 (containing 10 μg/mL OVA and 1 μg/mL R848) for 4 h in the absence of serum. For competition assay, the cells were pretreated with native mannan (5 mg/mL) for 1 h, followed by incubation with various formulations. To study the intracellular localization of OVA^Cy5^ or mOVA^Cy5^, lysosomes were stained with 50 nM LysoTracker Green, and the nuclei were stained with Hoechst 33258. Then, CLSM was used to observe the distribution of Cy5 signals in DC2.4 cells. Moreover, allergen uptake and binding by BMDCs were quantitatively evaluated using flow cytometry (BD Verse, BD Bioscience, USA). Immature BMDCs were plated into 24-well plates at 5 × 10^5^ cells per well, pretreated with or without mannan, and incubated with OVA^Cy5^, mOVA^Cy5^ or mOVA^Cy5^/R848 as mentioned above. After being washed twice with PBS, the BMDCs were resuspended in flow cytometry buffer and incubated with an anti-CD11c antibody before analysis by flow cytometry and FlowJo software (FlowJo 10.0.7, Tree Star, USA).

For maturation experiments, immature BMDCs were stimulated with OVA, mOVA, mOVA/R848 or R848 (containing 10 μg/mL OVA or 1 μg/mL R848) for 24 h 57. The BMDCs were harvested, resuspended in flow cytometry buffer, and incubated with antibodies against CD11c, CD40, CD86, MHC II, and CCR7 for 30 min before flow cytometry analysis.

### *In vitro* T cell differentiation

Single cell suspensions of spleens were collected from sensitized BALB/c mice, and lymphocytes were purified using the Spleen Lymphocyte Separation Kit. In detail, the spleens were mechanically fragmented into small pieces and gently squeezed through a 100 µm cell strainer. The resulting cell suspension was centrifuged, washed with a Spleen Lymphocyte Separation Kit, and resuspended in RPMI 1640 medium supplemented with 10% heat-inactivated FBS. Immature BMDCs were stimulated with OVA, mOVA, mOVA/R848 or R848 as described above. Then, they were cocultured with lymphocytes at a ratio of 1:10 for 72 h. T cell differentiation was analysed by flow cytometry. For Tregs, the harvested cells were incubated with Fixable Viability Dye eFluor™ 780 at 37 °C for 30 min. After washing, surface markers, including anti-CD4 and anti-CD25 antibodies, were stained at 4 °C for 30 min before being fixed with a Transcription Factor Buffer Set and stained with anti-Foxp3. For Th1 and Th2 cells, splenocytes from different groups were restimulated with leukocyte activation cocktail for 6 h before harvest. Then, the cells were stained with Fixable Viability Dye eFluor™ 780 at 37 °C for 30 min. After being washed twice with flow cytometry buffer, staining was performed against surface markers, including CD3 and CD4 antibodies, before being fixed with a Cytofix/CytoPerm Fixation/Permeabilization Solution Kit and stained with anti-IL-4 and anti-IFN-γ antibodies following the manufacturer's instructions.

The supernatant was collected and analysed for cytokines, including IL-4, IL-5, IL-13, IFN-γ, IL-12, TGF-β and IL-10, by ELISA kits according to the manufacturer's protocols.

### Kinetics of allergen capture and their migration to draining lymph nodes

To observe allergen persistence at the site of application and their migration to draining lymph nodes (dLNs), OVA or mOVA was labelled with Cy5-NHS ester according to the literature. The sensitized mice were randomly divided into six groups, and OVA pMN, mOVA pMN, mOVA/R848 pMN, mOVA/R848 dMN or R848 pMN arrays with 25 μg of OVA^Cy5^ and 2.5 μg of R848 were inserted into back skin with a self-made applicator. They were held in place manually for 15 min and secured with a bandage. At preset times, real-time imaging was recorded with an IVIS system in the Cy5 fluorescence channel and analysed with Living Image software (Perkin Elmer, USA).

For lymph node draining studies, the mice were sacrificed at days 2, 4 and 8, and images of the skin dLNs, including axillary and inguinal lymph nodes (LNs), were taken with the IVIS system at the Cy5 fluorescence channel. For flow cytometry analysis, freshly harvested LNs were gently grinded and filtered through 100 µm cell strainers. The resulting single cell suspensions were incubated with anti-CD11c, anti-CD40, anti-CD86 and anti-MHC II antibodies at 4 °C for 30 min to analyse the phenotypes of migrating DCs.

To study the penetration and distribution of allergens at the site of application, the skins of inoculation sites were dissected on the indicated days, embedded in optical cutting temperature (OCT) compound, and sectioned at a thickness of 10 μm. After being fixed with 4% paraformaldehyde, the sections were incubated with DAPI. Next, a slice scanner (SlideView VS200, Olympus, Japan) was used to visualize the mOVA^Cy5^ powder delivered into the skin.

### Safety profiles of cutaneous application

To assess the severity of erythema, the MN arrays were applied to mouse skin with a self-made applicator and then imaged at preset time points. Furthermore, haematoxylin and eosin (H&E) staining was used to assess epidermal and dermal inflammation.

### Immunotherapy schedule

The sensitized mice were randomly allocated into seven groups (n = 10): untreated, treated with MN arrays, or subjected to subcutaneous injections as further described. The proposed arrays, including the power-laden OVA pMN, mOVA pMN, mOVA/R848 pMN, R848 pMN and dissolving mOVA/R848 dMN patches with 25 μg of OVA and 2.5 μg of R848, were inserted into the back skin of the mice with a self-made applicator and secured with a bandage. As the hair grew quickly, the dorsal skin was prepared before each application as described above. These OVA-sensitized mice were treated biweekly for a total of three times. For subcutaneous immunotherapy (SCIT), the sensitized mice received a subcutaneous injection between the shoulders, with 200 μL of homogeneous suspension containing 25 μg of OVA and 0.25 mg of Alum. These mice were treated weekly for a total of six weeks. Moreover, sensitized mice without treatment (untreated) and healthy mice (naive) were also included.

Following treatment, all of the mice were challenged with aerosolized OVA for 30 min for three consecutive days. Blood samples were collected, and whole-body plethysmography (WBP) was performed. Additionally, the mice were sacrificed, and bronchoalveolar lavage fluids (BALFs), lung tissues and spleens were collected for the evaluation of local and systemic allergen-specific immune responses.

### Serological analysis

Blood samples were collected from the mouse eye socket into microtubes and centrifuged at 3000 rpm for 10 min to collect the serum. The serum levels of OVA-specific antibodies, including OVA-specific IgE, OVA-specific IgG1, and OVA-specific IgG2a (sIgE, sIgG1, and sIgG2a), were determined using a quantitative ELISA kit.

### Analysis of airway hyperresponsiveness and lung inflammation

Lung function was evaluated by monitoring airway responsiveness in response to aerosolized methacholine. Airway hyperresponsiveness (AHR) was performed 24 h after the last aerosol challenge via whole-body plethysmography (WBP-4M, Shanghai TOW Intelligent Technology, China). Pause enhancement (Penh) was recorded for 2 min after each methacholine stimulation (0, 3.125, 6.25, 12.5, 25, or 50 mg/mL). The Penh value is defined as the PEP/PIP, where the PEP is the peak expiratory pressure and the PIP is the peak inspiratory pressure.

BALFs were collected as follows. The mice were placed in a supine position after being deeply anaesthetized, and the trachea was exposed surgically. BALFs were obtained by syringe into the lungs by instilling and sucking 0.8 mL of PBS three times. Then, the obtained BALFs were centrifuged at 3000 rpm at 4 °C for 5 min. The supernatants were stored at -80 °C and used for cytokine analysis. The levels of cytokines (IL-4, IL-5, IL-13, TGF-β and IL-10) were determined by ELISA kits according to the manufacturer's recommendations.

The harvested lungs were cryosectioned and then stained with DHE (5 μM) for 30 min and DAPI (2 μg/mL) for 10 min. Next, fluorescence images were obtained using filters appropriate for excitation/emission = 518/605 nm. DHE is a superoxide anion (O_2-_) fluorescence detection probe for detecting oxidative activity in living cells to observe tissue reactive oxygen species (ROS) expression 58.

The harvested lungs were fixed in 4% paraformaldehyde, embedded, and cut into tissue sections. H&E staining was performed to observe morphological changes and infiltration of eosinophils and neutrophils. Periodic acid Schiff (PAS) staining was carried out to analyse the goblet cells and mucus deposition inside the bronchioles. Masson staining was used to analyse the deposition of collagen in the bronchioles. The bright-field images of the stained tissues were taken with a slice scanner as described above.

### Systemic immune response

The spleens were squeezed through a 100 μm cell strainer to obtain single cell suspensions. After RBC lysis, the cells were washed and resuspended in RPMI 1640 (supplemented with 10% heat-inactivated FBS). Subsequently, 1 × 10^6^ splenocytes were plated in 24-well plates and cultured with 100 μg/mL OVA. After 72 h of incubation, the supernatants were collected and then evaluated for IL-4, IL-5, IL-13, IFN-γ, IL-12, TGF-β and IL-10 concentrations using mouse ELISA kits. The treated splenocytes were harvested for evaluation of T-cell restimulation with OVA. For Tregs, the cells were incubated with Fixable Viability Dye eFluor™ 780, anti-CD4 and anti-CD25, before being fixed with a Transcription Factor Buffer Set and stained with anti-Foxp3. For analysis of Th1 and Th2 cells, the cells were incubated with leukocyte activation cocktail for 6 h before harvest. Then, the splenocytes from different groups were stained with Fixable Viability Dye eFluor™ 780, anti-CD3 and anti-CD4 antibodies, and fixed with a Cytofix/CytoPerm Fixation/Permeabilization Solution Kit, followed by staining with anti-IL-4 and anti-IFN-γ antibodies according to the manufacturer's instructions. The cells were analysed by flow cytometry and evaluated with FlowJo software.

For cell proliferation assay, 1 × 10^5^ spleen cells were cultured in 96-well plates and incubated with 100 μg/mL OVA or 5 μg/mL ConA as a positive control. After 72 h, cell proliferation in response to OVA was determined with a CCK-8 kit according to the manufacturer's instructions.

### Statistical analysis

All the data are presented as means ± standard deviations (SDs). One-way ANOVA was used for multiple comparisons when more than two groups were compared, whereas two-tailed Student's t test was used for two-group comparisons. Statistical significance is indicated as *P < 0.05, **P < 0.01, ***P < 0.001, and ****P < 0.0001.

## Supplementary Material

Supplementary figures and tables.
