# Supplementary material for: Co-delivery of targeted hypoallergens and resiquimod powders using silk fibroin microneedles for effective allergen-specific immunotherapy
Source: Theranostics. 2025 Jul 24;15(16):8096–116. doi: 10.7150/thno.114152 (PMC12374546; doi:10.7150/thno.114152)
Supplement: Supplementary file 1 — Supplementary figures and tables. [file thnov15p8096s1.pdf]

Co-delivery of targeted hypoallergens and resiquimod powders using silk fibroin microneedles for effective allergen-specific immunotherapy

Zhen Wang [a], Xiaolei Lu [a], Lingzhi Wu [a], Xin Jiang [a], Wei Wang [b, *], Yahua Wang [a, *]

[a] Department of Pharmacy, Affiliated Hospital of Jiaxing University, The First Hospital of Jiaxing, Jiaxing, 314000, China

[b] College of Materials and Textile Engineering, Jiaxing University, Jiaxing 314001, China

* Corresponding author.

E-mail:

Wei Wang: zjxuwangwei@163.com

Yahua Wang: 313717619@qq.com

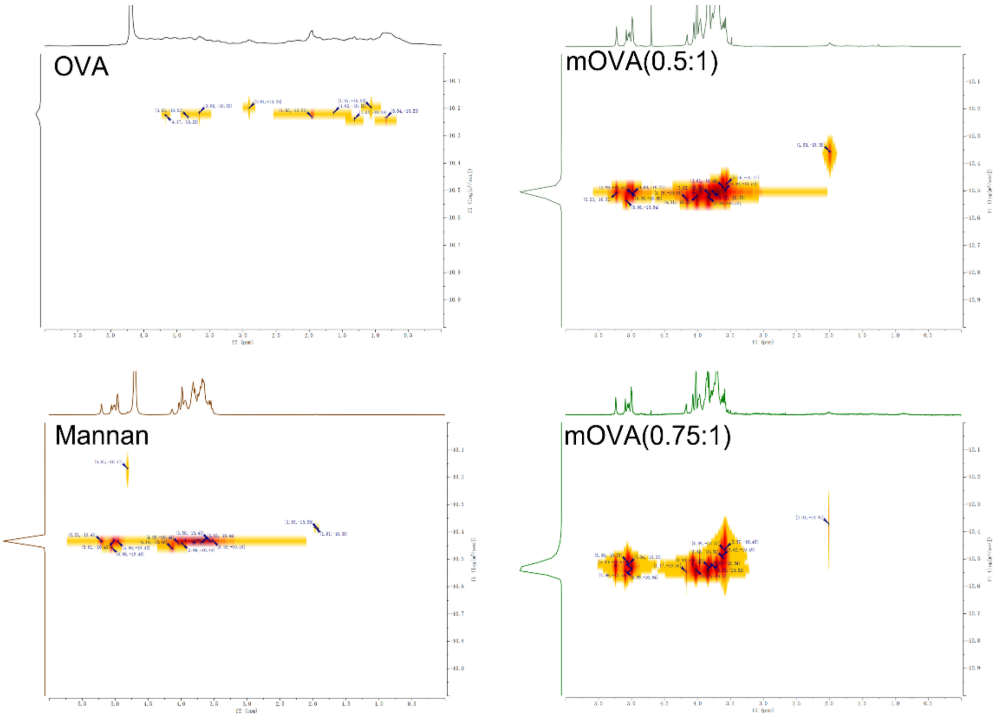

Figure S1. 2D-DOSY spectra for OVA, mannan and mOVA. OVA (black); Mannan (brown); mOVA (green): at ratio of 0.5: 1 and 0.75: 1.

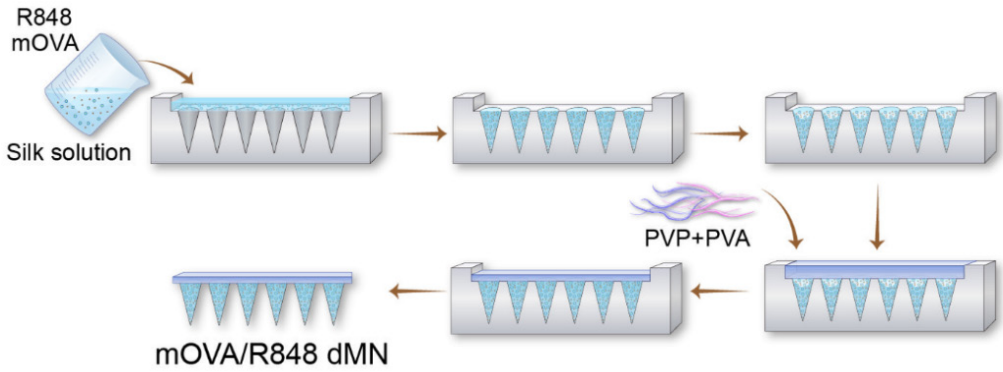

Figure S2. Fabrication of mOVA/R848 dMN arrays.

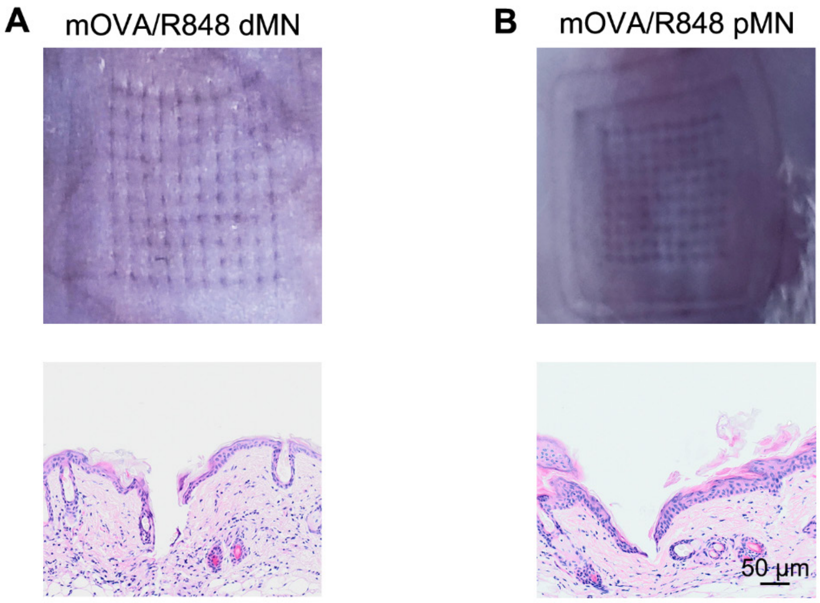

Figure S3. The holes created by mOVA/R848 dMN and mOVA/R848 pMN arrays on skin samples and their corresponding H&E staining results. Scale bar = 50 μm.

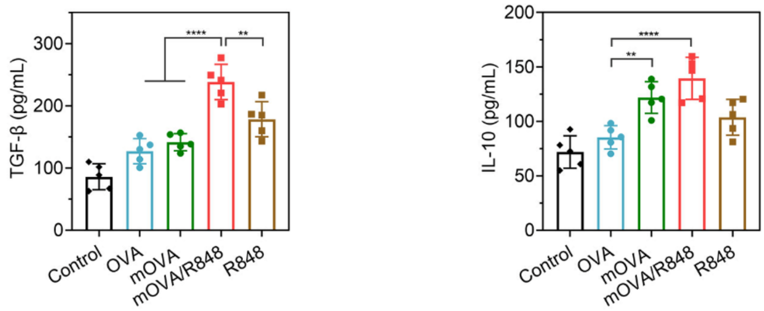

Figure S4. TGF-β and IL-10 from T cells activated by pretreated BMDCs. The data are presented as means ± SDs (n = 5). *$P < 0.05$, **$P < 0.01$, ***$P < 0.001$, and ****$P < 0.0001$.

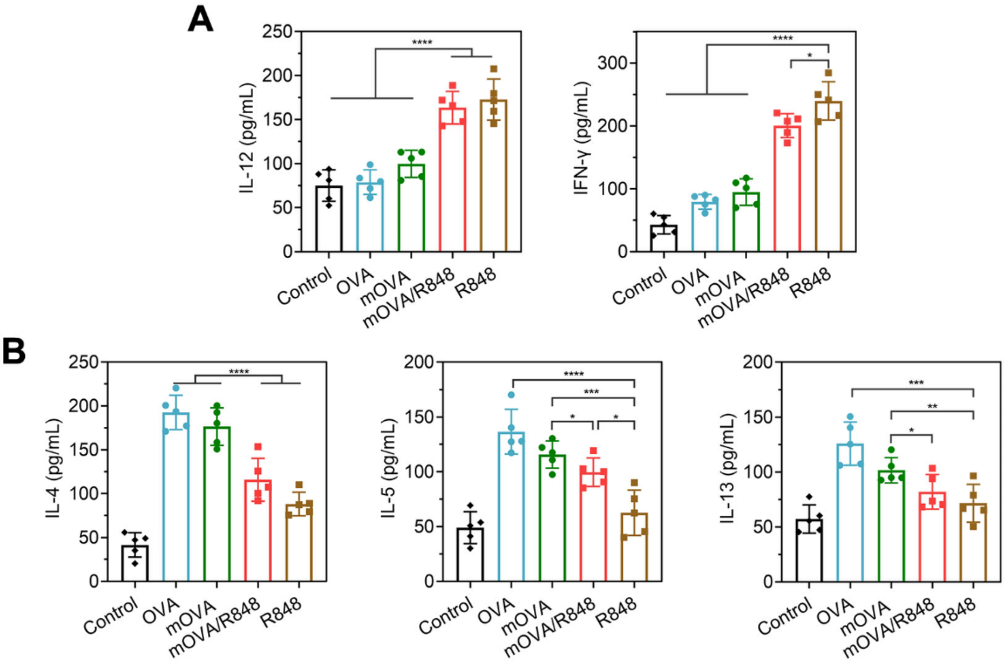

Figure S5. Secretion of (A) IL-12, IFN-γ and (B) IL-4, IL-5, IL-13 from T cells activated by pretreated BMDCs. The data are presented as means ± SDs (*n* = 5). **P* < 0.05, ***P* < 0.01, ****P* < 0.001, and *****P* < 0.0001.

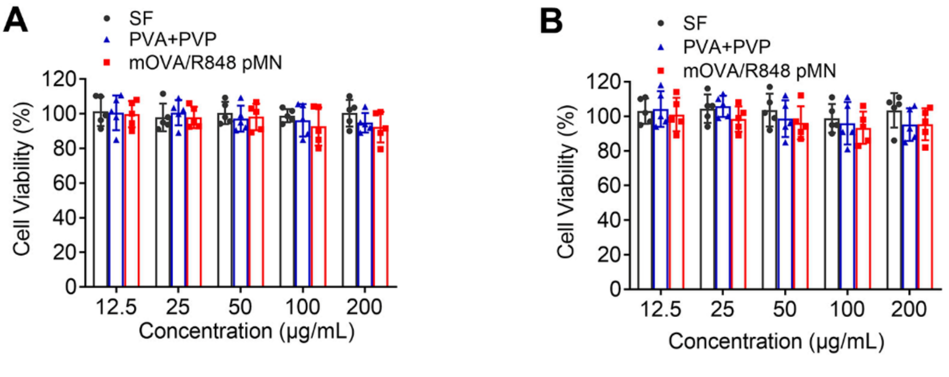

Figure S6. Cell proliferation with different extracting solutions. The data are presented as means ± SDs (n = 5).

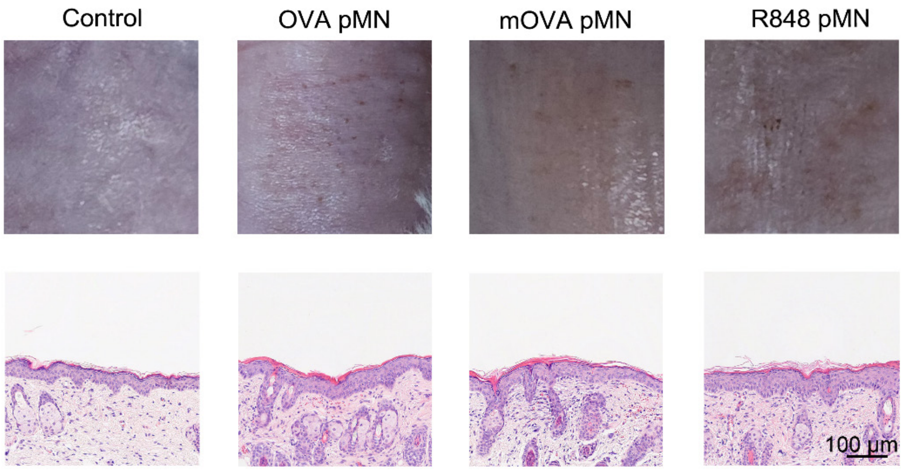

Figure S7. Representative images of skins at the treated site of mice and their corresponding H&E staining results. Scale bar = 100 μm.

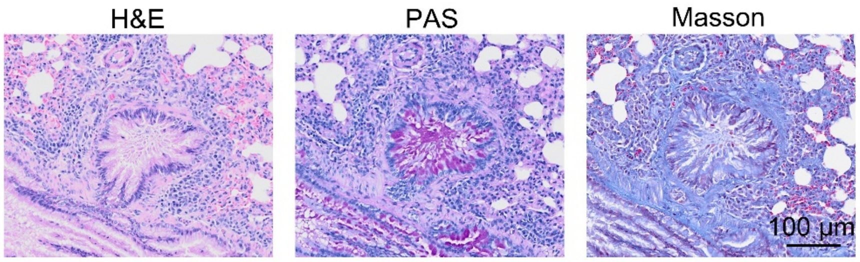

Figure S8. Representative H&E, PAS and Masson staining of lung sections for allergic asthma mice. Scale bar = 100 μm.

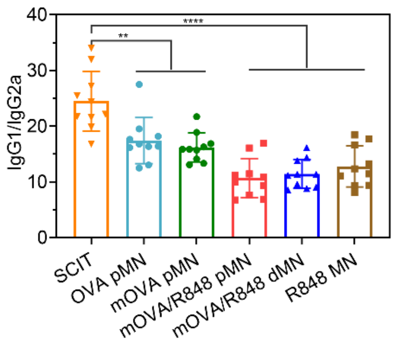

Figure S9. The ratio of OVA-specific IgG1 to OVA-specific IgG2a in serum. The data are presented as means ± SDs (n = 10). ***P < 0.01, *****P < 0.0001.

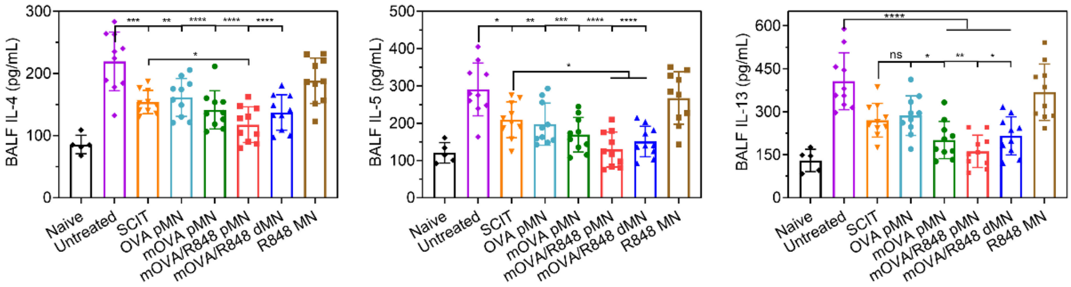

Figure S10. The levels of IL-4, IL-5, IL-13 in BALFs. The data are presented as means ± SDs (naive: n = 5; other: n = 10). *$P < 0.05$, **$P < 0.01$, ***$P < 0.001$, and ****$P < 0.0001$.

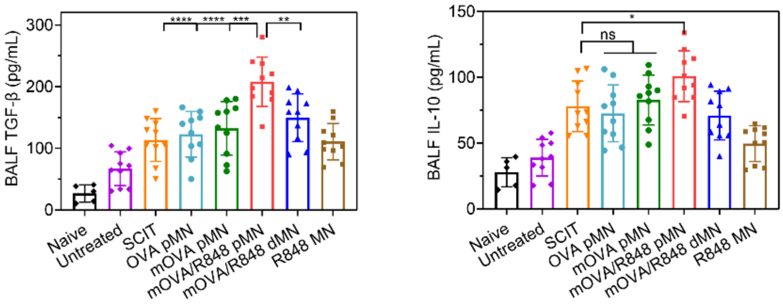

Figure S11. The levels of TGF-β and IL-10 in BALFs. The data are presented as means ± SDs (naive: n = 5; other: n = 10). *$P < 0.05$, **$P < 0.01$, ***$P < 0.001$, and ****$P < 0.0001$.

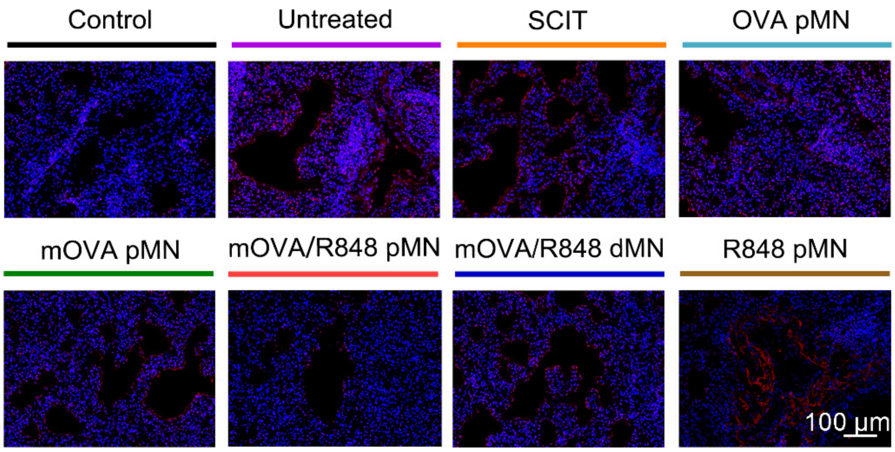

Figure S12. ROS in lung tissue was observed by DHE staining. Scale bar = 100 μm.

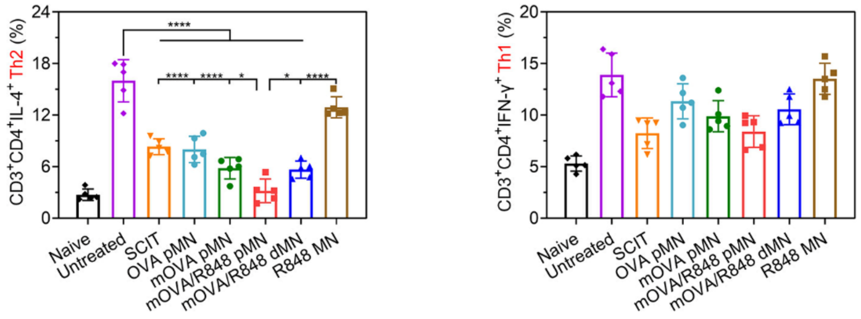

Figure S13. The proportion of Th2 and Th1 cells in splenocytes. The data are presented as means ± SDs (n = 5). *$P < 0.05$, **$P < 0.01$, ***$P < 0.001$, and ****$P < 0.0001$.

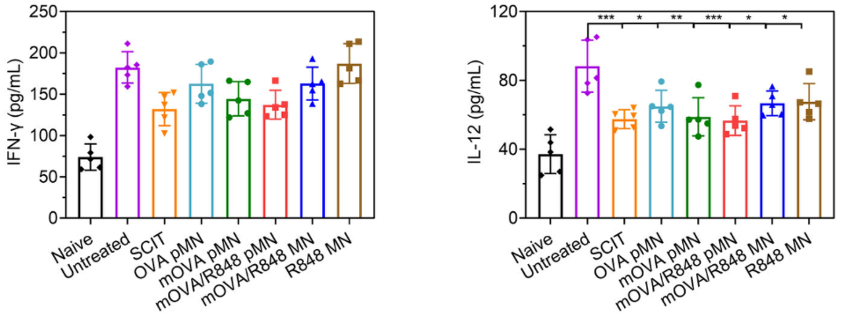

Figure S14. Secretion of IFN-γ and IL-12 from splenocytes. The data are presented as means ± SDs (n = 5). *$P < 0.05$, **$P < 0.01$, ***$P < 0.001$, and ****$P < 0.0001$.

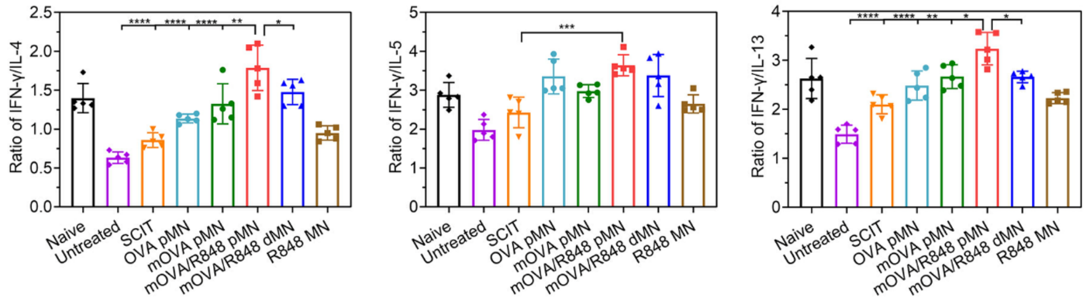

Figure S15. The ratios of IFN-γ/IL-4, IFN-γ/IL-5, IFN-γ/IL-13 in splenocytes. The data are presented as means ± SDs (n = 5). *$P < 0.05$, **$P < 0.01$, ***$P < 0.001$, and ****$P < 0.0001$.

Table S1. OVA and Mannan quantification in mOVA conjugates with different ratios. The data are presented as means ± SDs (n = 3).

Mannan (mg/mL)	OVA (mg/mL)	Feed Man/OVA ratio	Incorporated Man/OVA Ratio
5	10	0.5: 1	0.54: 1
7.5	10	0.75: 1	0.72: 1
10	10	1: 1	0.70: 1

Table S2. Loading capacity in MN arrays. The data are presented as means ± SDs (n = 3).

	OVA loading (μg)	R848 loading (μg)
OVA pMN	27.1 ± 1.2	
mOVA pMN	25.7 ± 2.8	
mOVA/R848 pMN	25.4 ± 3.0	2.3 ± 0.5
mOVA/R848 dMN	25.3 ± 2.5	2.1 ± 0.3
R848 pMN		2.8 ± 0.2

Table S3. Antibodies used in the study.

Antibody	Company	Clone	Catalog No.
CD11c (PE)	BioLegend	N418	177307
CCR7 (Alexa Fluor® 647)	BD Pharmingen	4B12	560766
MHC Class II (FITC)	eBioscience	M5/114.15.2	11-5321-81
CD40 (PerCP-eFluor™ 710)	eBioscience	1C10	46-0401-80
CD86 (PE-Cy7)	eBioscience	GL1	25-0862-80
CD3 (FITC)	eBioscience	145-2C11	11-0031-81
CD4 (PE)	BioLegend	GK1.5	100407
CD25 (PE-Cy7)	eBioscience	PC61.5	25-0251-81
FOXP3 (Alexa Fluor® 647)	BD Pharmingen	MF23	560401
IL-4 (PE-Cy7)	eBioscience	11B11	25-7041-80
IFN-γ (APC)	eBioscience	XMG1.2	17-7311-81
Fixable Viability Dye eFluor™ 780	eBioscience		65-0865-14

Table S4. ELISA kits used in the study.

ELISA kits	Company	Catalog No.
OVA-sIgE	Yifeixue Bio Tech	YFXEM00075
OVA-sIgG1	Yifeixue Bio Tech	YFXEM00656
OVA-sIgG2a	Yifeixue Bio Tech	YFXEM00657
IL-4	Yifeixue Bio Tech	YFXEM00057
IL-5	Yifeixue Bio Tech	YFXEM00012
IL-13	Yifeixue Bio Tech	YFXEM00013
IL-10	Yifeixue Bio Tech	YFXEM00065
TGF-β	Yifeixue Bio Tech	YFXEM00561
IFN-γ	Yifeixue Bio Tech	YFXEM00039
IL-12	Yifeixue Bio Tech	YFXEM00599
